# Disrupting Na^+^ ion homeostasis and Na^+^/K^+^ ATPase activity in breast cancer cells directly modulates glycolysis in vitro and in vivo

**DOI:** 10.1186/s40170-024-00343-5

**Published:** 2024-05-24

**Authors:** Aidan M. Michaels, Anna Zoccarato, Zoe Hoare, George Firth, Yu Jin Chung, Philip W. Kuchel, Ajay M. Shah, Michael J. Shattock, Richard Southworth, Thomas R. Eykyn

**Affiliations:** 1 School of Biomedical Engineering and Imaging Sciences, King’s College London, St Thomas’ Hospital, London, SE1 7EH UK; 2 School of Cardiovascular and Metabolic Medicine and Sciences, King’s College London, London, UK; 3 School of Life and Environmental Sciences, University of Sydney, Sydney, NSW 2006 Australia

**Keywords:** Breast cancer, Glycolysis, Intracellular sodium, NaK ATPase, Ouabain, Warburg effect

## Abstract

**Background:**

Glycolytic flux is regulated by the energy demands of the cell. Upregulated glycolysis in cancer cells may therefore result from increased demand for adenosine triphosphate (ATP), however it is unknown what this extra ATP turnover is used for. We hypothesise that an important contribution to the increased glycolytic flux in cancer cells results from the ATP demand of Na^+^/K^+^-ATPase (NKA) due to altered sodium ion homeostasis in cancer cells.

**Methods:**

Live whole-cell measurements of intracellular sodium [Na^+^]_i_ were performed in three human breast cancer cells (MDA-MB-231, HCC1954, MCF-7), in murine breast cancer cells (4T1), and control human epithelial cells MCF-10A using triple quantum filtered ^23^Na nuclear magnetic resonance (NMR) spectroscopy. Glycolytic flux was measured by ^2^H NMR to monitor conversion of [6,6-^2^H_2_]d-glucose to [^2^H]-labelled l-lactate at baseline and in response to NKA inhibition with ouabain. Intracellular [Na^+^]_i_ was titrated using isotonic buffers with varying [Na^+^] and [K^+^] and introducing an artificial Na^+^ plasma membrane leak using the ionophore gramicidin-A. Experiments were carried out in parallel with cell viability assays, ^1^H NMR metabolomics of intracellular and extracellular metabolites, extracellular flux analyses and in vivo measurements in a MDA-MB-231 human-xenograft mouse model using 2-deoxy-2-[^18^F]fluoroglucose (^18^F-FDG) positron emission tomography (PET).

**Results:**

Intracellular [Na^+^]_i_ was elevated in human and murine breast cancer cells compared to control MCF-10A cells. Acute inhibition of NKA by ouabain resulted in elevated [Na^+^]_i_ and inhibition of glycolytic flux in all three human cancer cells which are ouabain sensitive, but not in the murine cells which are ouabain resistant. Permeabilization of cell membranes with gramicidin-A led to a titratable increase of [Na^+^]_i_ in MDA-MB-231 and 4T1 cells and a Na^+^-dependent increase in glycolytic flux. This was attenuated with ouabain in the human cells but not in the murine cells. ^18^FDG PET imaging in an MDA-MB-231 human-xenograft mouse model recorded lower ^18^FDG tumour uptake when treated with ouabain while murine tissue uptake was unaffected.

**Conclusions:**

Glycolytic flux correlates with Na^+^-driven NKA activity in breast cancer cells, providing evidence for the ‘centrality of the [Na^+^]_i_-NKA nexus’ in the mechanistic basis of the Warburg effect.

**Supplementary Information:**

The online version contains supplementary material available at 10.1186/s40170-024-00343-5.

## Introduction

Glycolysis is an evolutionarily conserved metabolic pathway that oxidises one molecule of glucose to form two molecules of pyruvate, typically producing a net of two molecules of ATP. The product pyruvate can either enter the tricarboxylic acid (TCA) cycle in the mitochondria, where it is further oxidized, phosphorylating ~ 32 more molecules of adenosine diphosphate (ADP); or it is reduced to lactate with conversion of NADH to NAD^+^ (nicotinamide adenine dinucleotide) that is recycled as a co-substrate of glyceraldehyde-3-phosphate dehydrogenase (GAPDH), thus enabling glycolysis to continue; or it is transaminated to form alanine. In normal cells, increased glycolytic flux is typically observed under anaerobic conditions where oxygen supply is not able to meet demand and oxidative phosphorylation is inhibited in the mitochondria, the build-up of lactate and H^+^ is prevented by efflux via plasma membrane monocarboxylate transporter protein(s). The Warburg effect [1–3] is a phenomenon in which many cancer cell types (although not all) preferentially use glycolysis, even in the presence of plentiful oxygen. The premise of a ‘glycolytic switch’ as the basis of this effect can be misleading, as it has been extensively reported that cancer cells have fully functional mitochondria [4–7], contrary to Warburg’s initial thesis. However, cancer cells are reported to have slower TCA cycle flux than healthy cells [8]. Therefore, a fundamental question remains: Why do cancer cells have increased glycolytic metabolism and what is the extra ATP used for? This paradoxical observation, in the sense that uncontrolled growth of cells would require the most efficient extraction of energy from metabolic fuels like glucose, would imply up-regulation of oxidative phosphorylation, not the reverse [9]. It has often been argued that the switch in metabolism in cancer cells favours the accumulation of biomass since increased flux through glycolysis supports metabolic shunts such as the pentose phosphate pathway (PPP) and various branch pathways leading to the synthesis of amino acids required for protein synthesis [10]. However, recent suggestions are that glycolysis supplies the ATP required to satisfy the fluctuating anabolic demands of the cell [11], rather than being used to maintain a steady state metabolite concentration, and that glycolysis directly provides the ATP for other energy-demanding processes like the maintenance of the trans-plasma-membrane ionic gradient [12].

The enzyme-catalysed reactions of glycolysis transduce covalent-bond energy from glucose to form ATP (from ADP and inorganic phosphate); and it is known that flux through the pathway is ‘ATP-demand regulated’ [13]. For the cell to remain in a metabolic steady-state the number of moles of ATP generated must match the number of moles of ATP consumed in any time interval, by processes separate from the glycolytic reactions [11]. Hence, an increase in the rate of ATP hydrolysis will release more ADP and increase the [ADP]/[ATP] ratio; this results in the allosteric stimulation of glycolysis via phosphofructokinase-1 (PFK-1), producing more ATP to match demand [14].

The major cytosolic sinks for ATP are Na^+^/K^+^-ATPase (NKA), Ca^2+^-ATPases, H^+^/K^+^-ATPase, and H^+^-ATPases which belong to the superfamily of ion pumps known as P-type ATPases [15]. Of these NKA is reported to be responsible for ~ 30% of the total cellular ATP turnover with some estimates as much as 50% of total ATP turnover in the brain [16]. In the human erythrocyte, which lacks mitochondria, ATP generation is entirely glycolytic, and it has previously been shown through inhibition of the NKA with ouabain, or stimulation of Na^+^ influx with the ionophore monensin, that the stoichiometry of Na^+^ efflux via NKA is close to the theoretical 6 ions of Na^+^per 1 molecule of glucose consumed [17]. This highlights the intimate connection between the activity of the pump and glycolytic flux in these cells. A tight coupling between glycolytic metabolism and NKA activity has been reported in renal MDCK cells [18], permeabilized rat cardiomyocytes [19], and Ehrlich ascites tumour cells [14]. Thus, increased activity of NKA would increase ATP hydrolysis which in turn stimulates glycolytic production of ATP that matches demand. However, it is not known to what extent this process also drives the high glycolytic rates typically seen in cancer cells.

Na^+^ion homeostasis is widely reported to be dysregulated in cancer [20], being elevated inside the cell [21] and tumours [21–23]. Increased intracellular Na^+^ could either result from decreased activity of the NKA, the main extruder of Na^+^ from the cell, or an increase in Na^+^influx [24, 25]. Observations that Na^+^-channels are overexpressed in cancer cells [25, 26] suggests that increased influx may be the major driver of elevated [Na^+^]_i_, which is consistent with the overexpression of sodium channel protein type 5 subunit alpha (Na_V_1.5) [27] and other voltage gated Na^+^-channels [28] being associated with more rapid disease progression and worse patient outcomes [29]. Other Na^+^influx pathways include sodium hydrogen exchange (NHE) and sodium bicarbonate co-transport (NBC), which are linked to cytosolic pH through the action of carbonic anhydrases (CAs) [30], as well as sodium glucose linked transport (SGLT) where Na^+^ transport is coupled to glucose uptake. Independent of the mechanism of Na^+^ influx, a consequence of increased intracellular [Na^+^] would be to push NKA up its activation curve (i.e., above its K_m_ for Na^+^), thereby increasing ATP turnover by NKA. A further consequence of elevated [Na^+^]_i_ will be an elevation of cytosolic Ca^2+^ through the action of the plasma membrane sodium-calcium exchange (NCX) and a decrease of mitochondrial Ca^2+^ through the action of the mitochondrial sodium-calcium exchange (NCLX) which lead to wide ranging effects of altered Na^+^ ion homeostasis in cancer cells.

We focussed on the contribution of NKA to glycolytic metabolism and hypothesised that the elevated rates of glycolysis that underpins the Warburg effect in cancer is causally linked to the flux of Na^+^ through the NKA. We tested this hypothesis in a panel of three human breast cancer cells MDA-MB-231, HCC1954, MCF-7, a murine breast cancer cell line 4T1, and a control human epithelial cell line MCF-10A. Nuclear magnetic resonance (NMR) techniques were applied to living cells, to probe [Na^+^]_i_ non-invasively with triple quantum filtered (TQF) [23] ^23^Na-NMR spectroscopy [31] and glycolytic flux measurements with ^2^H-NMR to measure conversion of [6,6-^2^H_2_]d-glucose to [^2^H]-labeled l-lactate, in real time, and in response to NKA inhibition with ouabain [32]. Intracellular [Na^+^]_i_ was titrated using modified isotonic extracellular buffers with varying [Na^+^] and [K^+^] and introducing an artificial Na^+^ plasma membrane leak using the ionophore gramicidin-A, to create a controlled and constant permeabilization to Group 1 cations, which equilibrates [Na^+^] across the cell membrane. Experiments were carried out in parallel with cell viability assays, ^1^H NMR metabolomics of intracellular and extracellular metabolites, extracellular flux analyses using the commercial Seahorse assay method to measure extracellular acidification rate (ECAR) and oxygen consumption rate (OCR), and in vivo measurements in an MDA-MB-231 human-xenograft mouse model using 2-deoxy-2-[^18^F]fluoroglucose (^18^F-FDG) positron emission tomography (PET) [33].

## Materials and Methods

### Cell culture and treatment protocols

Human cell lines MDA-MB-231 (CRM-HTB-26); MCF-7 (HTB-22); HCC1954 (CRL-2338) and 4T1 (CRL-2539) mouse cell line were obtained from American Type Culture Collection (ATCC). MCF-10A (CRL-10317) were a gift from Dr Christopher Switzer. All cells were used at early passage and tested regularly for mycoplasma. Cells were cultured as monolayers under standard conditions in the supplier’s recommended media: RPMI-1640 (#R0883-500ML, Sigma, USA), DMEM (#D5671-500ML, Sigma, USA); DMEM/F-12 (#11,330–032, Gibco). RPMI-1640 and DMEM were supplemented with 10% (v/v) fetal bovine serum (FBS) (#F7524-500ML, Sigma-Aldrich), 4 mM L-glutamine (#G7513-100ML, Sigma-Aldrich), 170 µM penicillin and 170 µM streptomycin (#P4333-100ML, Sigma-Aldrich). DMEM/F-12 (Gibco) was supplemented with 5% (v/v) horse serum (26,050,088, ThermoFisher Scientific) 10 µg mL^−1^ human insulin (A11382II, Gibco), 20 ng mL^−1^ human epidermal growth factor (hEGF) (#CC-4107, Lonza), 100 ng mL^−1^ cholera toxin (#C8052, Sigma-Aldrich), 0.5 µg mL^−1^ hydrocortisone (H0888, Sigma-Aldrich).

For investigations using ouabain, normal medium was replaced 1 h prior to the start of the experiment with complete DMEM supplemented with 1 µM ouabain (#PHR1945, Sigma-Aldrich). For control MCF-10A cells, complete medium was replaced 16 h prior to experimentation by DMEM/F-12 solely supplemented with 5% (v/v) horse serum to avoid artificial growth stimulation.

### Cell volume measurement

Cell volumes were measured using a Scepter 2.0 Automatic cell counter (#PHCC20040, Merck Millipore, USA) with Scepter™ 60 µm sensors attached (#PHCC60050, Merck Millipore, USA). Samples were taken as part of routine passaging during experiments and assessed in serum-free DMEM supplemented with 4 mM L-glutamine, 170 µM penicillin and 170 µM streptomycin. The mean cell volume (pL) was used to calculate the average cell size, which was used for normalization throughout (data in Supplementary Fig. S1).

### MTT cytotoxicity assays

Cells were seeded at 1 × 10^5^ cells mL^−1^ into a 96-well plate. Following overnight adherence, the cells were cultured with complete medium ± ouabain (titrated 1 × 10^–12^ – 1 × 10^–3^ M) for 24 h. The medium was then aspirated and replaced with 20 μL solution of reconstituted powder 3-(4,5-dimethylthiazol-2-yl)-2,5-diphenyltetrazolium bromide (MTT; #M6494, ThermoFisher Scientific) in each well and mixed. After 3 h, supernatants were removed and 50 μL dimethyl sulfoxide (DMSO) was added to each well to dissolve the formazan crystal precipitate. Light absorbance was measured at a wavelength of 570 nm using a Spectrostar Nano plate reader (BMG LabTech, Germany). Data were fitted using a nonlinear regression model in GraphPad Prism version 9.4.1 for Windows (GraphPad Software, USA) to estimate EC_50_ following 24 h ouabain treatment. Normalised absorbance is presented as a % of maximum.

### Trypan blue exclusion

Cells were seeded at densities of 5 × 10^5^ per well in 2 mL of complete medium and left to adhere overnight. The following day, the medium was aspirated and replaced with DMEM ± 1 µM ouabain for 1 h. Medium was aspirated, and the cells were washed three times with 1 mL Dulbecco’s phosphate buffered saline (DPBS; #D8537, Sigma Life Science), 0.05% Trypsin–EDTA solution (#15,400–054, Gibco) was added to each well until the cells detached. The cells were resuspended in serum-free DMEM and centrifuged at 350 × g for 5 min. Cell pellets were resuspended in serum-free DMEM and diluted to give concentrations of 5 × 10^4^ cells mL^−1^ and vortexed to ensure a homogeneous suspension. Fractions of each cell suspension were mixed 1:1 with 0.4% Trypan Blue solution (#T8154, Sigma Life Sciences) and pipetted onto a haemocytometer. Studies were performed within 5 min of mixing with Trypan Blue. Cell viability was calculated by $\text{Cell viability}=(\text{Viable cells}/\text{Total cells})\times 100\%$. Unpaired, two-tailed t-tests were performed in GraphPad Prism version 9.4.1 for Windows (GraphPad).

### ^1^H NMR metabolomics

Cells were harvested by scraping the surface and then subjecting to methanol / water / chloroform dual phase extraction. Cells were vortexed in 2 mL each of ice-cold methanol, chloroform, and Millipore water and then centrifuged for 1 h at 2500 × g at 4 °C to separate aqueous, protein, and lipid layers. The upper aqueous phase was separated; 20–30 mg Chelex-100 was added to chelate paramagnetic ions, vortexed and centrifuged at 2500 × g for 1 min at 4 °C. The supernatant was transferred to a fresh Falcon tube containing 10 μL of universal pH indicator solution followed by freeze drying. Dual-phase-extracted metabolites were reconstituted in 600 μL of deuterium oxide [containing 8 g L^−1^ NaCl, 0.2 g L^−1^ KCl, 1.15 g L^−1^ Na_2_HPO_4_, 0.2 g L^−1^ KH_2_PO_4_ and 0.0075% w/v 3-(trimethylsilyl)-2,2,3,3-d_4_-tetradeuteropropionic acid (TSP)] and adjusted to pH ~ 7 by titrating with 100 mM hydrochloric acid. Cell culture medium samples (500 µL) were collected from the culture flask after 24 h. Fresh medium samples were also collected. 100 µL of D_2_O buffer were added to each sample prior to NMR spectral acquisition.

^1^H nuclear magnetic resonance spectra were acquired using a vertical-bore, ultra-shielded Bruker (Bruker, Karlsruhe, Germany) 14.1 T (600 MHz) spectrometer equipped with a prodigy probe, at 298 K using the Bruker noesygppr1d pulse sequence for residual water suppression. Acquisition parameters were: 64 transients; 4 dummy transients; 20.8 ppm spectral width; acquisition time, 2.6 s; pre-scan delay, 4 s; 90° flip angle; and experiment duration of 7.5 min per sample. TopSpin (version 4.0.5) software was used for data acquisition and for metabolite quantification. Free induction decays (FIDs) were multiplied by a line broadening factor of 0.3 Hz and Fourier transformed, phase, and automatic baseline corrected. Chemical shifts were normalised by setting the TSP signal to 0.0 ppm. Metabolite peaks of interest were initially integrated automatically using a pre-written integration-region text file and then manually adjusted as required. Assignment of peaks to their respective metabolites was based on previously obtained in-house data, confirmed by chemical shift, and using Chenomx NMR Profiler Version 8.1 (Chenomx, Canada). Metabolite concentrations were normalised to the number of cells (measured in a parallel flask) and cell volume, expressed in mM.

### ^23^Na triple quantum filtered NMR

Cells were seeded 48 h prior to experiments to reach 70–80% confluence at the time of harvesting. For ouabain treatment groups the medium was replaced 1 h prior to harvesting. Monolayer cultures were harvested by trypsinisation, resuspended in DMEM without 10% FBS, counted, and their viability assessed by the trypan blue exclusion method. Cell suspensions were centrifuged at 350 × g for 5 min at room temperature. The supernatant was aspirated, and the cell pellet was rapidly resuspended in 250 µL DMEM without 10% FBS. The cell suspension was transferred to a 5 mm OD Shigemi NMR tube, 8 mm bottom glass slug, (D_2_O matched #BMS-005B, Fluorchem) containing a 20-mm long, 1-mm OD capillary tube that contained 2% w/v agarose (#17,850, ThermoFisher Scientific), 5 mM 1,4,7,10-tetraazacyclododecane-1,4,7,10-tetramethylenephosphonate (Tm[DOTP]^5−^; #M-155, Macrocyclics, Plano TX, USA) without using a deuterium-solvent lock. The NMR tube was then placed in a Bruker Avance III 400 MHz wide-bore spectrometer with a BBO probe maintained at 310 K (37 °C). Cells were transferred from their culture medium and studied by NMR within 10 min. Magnetic field homogeneity was ensured by shimming on the 1D ^23^Na spectrum prior to each experiment. Triple quantum filtered ^23^Na NMR experiments were performed as previously described [31, 34], with acquisition of 384 transients, 6 dummy transients, a spectral width of 50 ppm, pre-scan delay of 150 ms, and an acquisition time of 2 min 16 s. TQF transients were repeated three times in succession to assess reproducibility.

### ^2^H NMR of glycolytic flux

Cell samples were cultured, harvested, and prepared as described above. Cell pellets were resuspended in 250 µL glucose-free DMEM (#11,966,025, ThermoFisher Scientific) and transferred to a 5 mm OD Shigemi NMR tube. A 40 µL bolus of [6,6-^2^H_2_]d-glucose (#282,650, Sigma-Aldrich) was added to give a final concentration of 10 mM D-glucose within the sample. The cell suspension was then placed in the Bruker Avance III 400 MHz wide-bore spectrometer, with a BBO probe (Bruker) maintained at 37 °C for the duration of the experiment. To improve sensitivity in the detection of ^2^H, the X transmitter amplifier was routed via the ^2^H preamplifier, and onto the X-channel of the 5-mm BBO probe. Time series of spectra were acquired in a pseudo-2-dimensional fashion using inverse gated ^1^H decoupling (waltz16), with 128 transients, a spectral width of 20 ppm, a pre-scan delay of 10 ms, and an acquisition time of 200 ms. Each spectrum was averaged over 28.4 s with 64 spectra acquired in a time course of ~ 30 min. Spectra were processed and analysed using TopSpin 4.2.0 (Bruker). Peak integrals were measured for [^2^H]HOD, [6,6-^2^H_2_]d-glucose and [3,3-^2^H_2_]l-lactate as a function of time. The dynamic intensity versus time data were analysed using nonlinear least squares fitting to a two-compartment model in MATLAB R2022b (Mathworks, USA) to the estimate rate constant for the conversion of glucose to lactate. Briefly, a phenomenological model given by the following set of differential equations was employed; it assumed a single rate constant *k*_*gl*_ for glucose to lactate conversion and an additional rate constant *k*_*loss*_ describing flux of glucose via other pathways,

1 $$\frac{dGlu(t)}{dt}=-{k}_{gl}Glu\left(t\right)-{k}_{loss}Glu\left(t\right)$$

2 $$\frac{dLac(t)}{dt}={k}_{gl}Glu\left(t\right)$$

Experimental data were fitted in a least squares fashion using an ordinary differential equation (ODE) solver. Glycolytic rate constants (*k*_*gl*_) were multiplied by the number of moles of glucose added, and normalised to cell number, and cell volume (Supplementary Fig. S1) to give glycolytic flux in units of mol (total cell volume)^−1^ s^−1^.

### Extracellular Flux

Extracellular acidification rate (ECAR) was measured with an XFe Extracellular Flux Analyzer (Agilent, USA) that was run according to the manufacturer’s instructions. Cells were seeded at 5 ✕ 10^4^ cells per well, plated on Seahorse XFe24 culture plates (Agilent, #100,777–004) in the preferred medium (RPMI-1640 or DMEM) supplemented with 10% (v/v) FBS, 4 mM L-glutamine, 170 µM penicillin, and 170 µM streptomycin, and incubated overnight.

Two hours prior to the start of the experiment, the culture medium was replaced with DMEM ± 1 µM ouabain for 1 h and then placed into standard culture conditions. The medium was aspirated, and replaced with unbuffered DMEM (#D5030, SigmaAldrich) supplemented with 4 mM L-glutamine, pH 7.4, and incubated for 1 h at 37 °C in a CO_2_-free incubator. The glycolysis stress-test was then performed according to Seahorse commercial protocols with final concentrations of 10 mM glucose, 1 μM oligomycin, and 100 mM 2-deoxy-d-glucose. The ECAR reading was normalised to DNA content in each well using DRAQ5 nuclear stain (Thermo Scientific, #62,251) at the end of the experiment. Full traces of the glycolytic stress test were presented, including all repeats ± SD.

### Gramicidin

Cells were prepared in 175 cm^2^ culture flasks and seeded 36 h prior to each experiment (80–90% confluence). Cell culture medium was aspirated and replaced with complete DMEM ± 1 µM ouabain for 1 h. The medium was then aspirated and the cells were washed with DPBS. Trypsin–EDTA was used to detach the cells, and the medium was neutralised by adding modified Krebs–Henseleit buffer (Supplementary Table S1) not containing Na^+^. Cell suspensions were centrifuged at 350 ✕ g for 5 min. The supernatant was aspirated and the cells were resuspended in 250 µL of the respective buffers in (mM) 10, 20, 30, 50 or 70 [Na^+^] containing 10 µM Gramicidin (#G5002, Sigma-Aldrich) dissolved in sterile dimethyl sulfoxide (#12,611, Cell Signalling Technology) and transferred to a 5-mM OD Shigemi NMR-tube. To this tube either the Tm-DOTP reference tube was added for ^23^Na- TQF NMR experiments or 10 mM [6,6-^2^H_2_]d-glucose was added for ^2^H NMR experiments. The sample was then subject to the same NMR experiments for acquisition of either ^23^Na TQF spectra to measure [Na^+^]_i_ or ^2^H time series to measure glycolytic flux.

### Animal models

All animal experiments were conducted in the UK and were performed in accordance with the United Kingdom Home Office Animal (Scientific Procedures) Act, 1986. Experiments were performed unblinded. BALB/c Nude (CAnN.Cg-Foxn1nu/Crl) were purchased from Charles River Laboratories. Mice arrived at age 6–8 weeks and were acclimatized for 7 d before undergoing experimental procedures. Animals were able to access drinking water and standard diet ad libitum.

### ^18^F-FDG PET preclinical imaging

Tumours were established by injecting 100 µL of suspension containing 1 × 10^6^ MDA-MB-231 cells in a 1:1 PBS/Matrigel (#AC-M082706, Acro Biosystems) solution. Tumour growth was monitored daily by electronic calliper measurement with volume calculated using the following equation: volume (mm^3^) = ((π/6) × h × w × l), where h, w and l represent height, weight, and length, respectively. When tumour volumes reached > 100 mm^3^, animals underwent PET imaging protocols. At t = 0, sterile saline ± 0.04 mg kg^−1^ ouabain was injected via tail vein cannulation as a 50 µL bolus to anaesthetised mice. The injected concentration of ouabain was 270 µM to yield a final blood concentration of ~ 6.75 µM; this was above the EC_50_ measured for human MDA-MB-231 cells but below the EC_50_ for mouse cells. Mice were anaesthetised with isoflurane (2–2.5% in O_2_) by nose cone at 37 °C until transferred to the nanoScan® microPET/CT scanner (Mediso). At t = 60 min, mice were injected with 7.15 ± 1.33 MBq ^18^F-FDG in saline (n = 12), and a dynamic PET acquisition was performed for 1 h post injection. After completion of scanning protocols, mice were euthanized by cervical dislocation. For quantification of radiotracer retention in tissue by PET/CT, tumour volumes were estimated from 2D regions drawn manually using the CT image as reference using VivoQuant™ (Invicro, Needham, MA, USA). Data were expressed as %ID g^−1^, assuming a tissue density of d = 1 g mL^−1^.

### Statistics

**Fig. 1:**
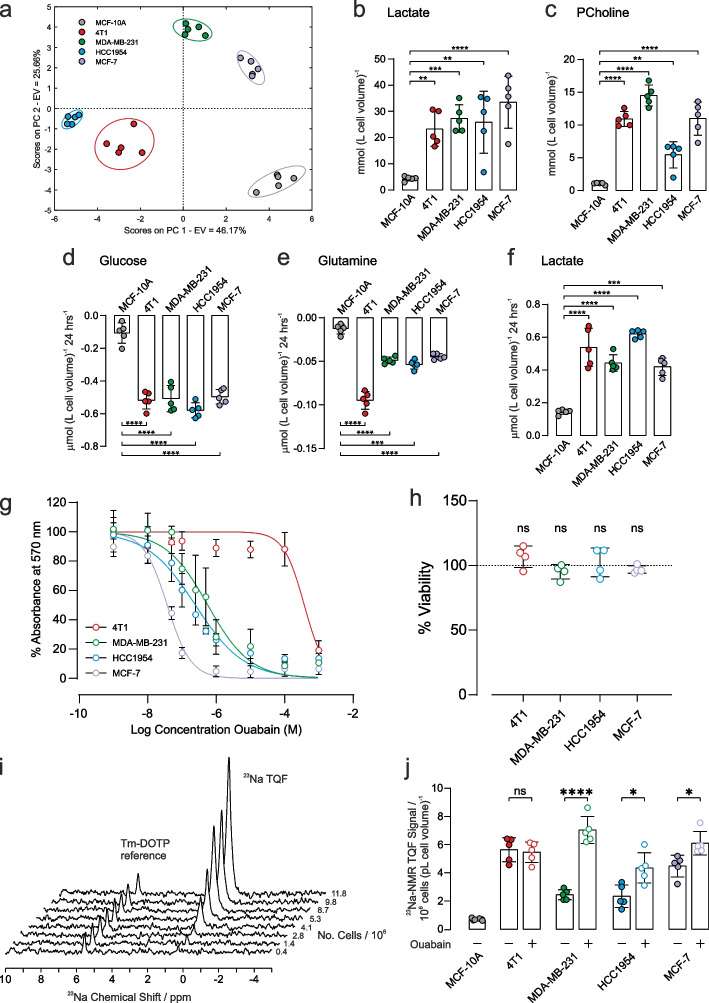
Metabolic characterization and response to ouabain treatment on [Na^+^]_i_. **a** Principal component analysis of intracellular metabolites (mean values given in Supplementary Table S2). Intracellular concentrations of **b** lactate (left panel) and **c** phosphocholine (right panel) were significantly higher in all cancer cells with respect to control epithelial cells (*n* = 5). Extracellular metabolite concentrations of **d** glucose, **e** glutamine and **f** lactate after 24 h cell culture. Respective metabolite concentration from fresh media were subtracted such that negative concentrations refer to metabolite consumption while positive concentrations refer to production (*n* = 5). **g** MTT cytotoxicity assay dose response curves following 24 h treatment with ouabain. Measured EC_50_ values were: 4T1: 40 µM, MDA-MB-231: 0.4 µM, HCC1954: 0.2 µM, MCF-7: 0.04 µM. **h** Cell viability in response to 1 µM ouabain for 1 h measured by trypan blue exclusion assay measured no change in cell viability. **i** Representative TQF ^23^Na NMR spectra showing proportionality with cell number. The Tm-DOTP reference peak is from the internal standard. **j** Quantification of TQF ^23^Na NMR relative to cell number and cell volume. Baseline [Na^+^]_i_ was higher in all cancer cells with respect to control epithelial cells (*n *= 5). Treatment with 1 µM ouabain for 1 h led to a significant increase in [Na^+^]_i_ in all human cancer cell lines compared to vehicle control (*n* = 5). [Na^+^]_i_ was unchanged in the murine 4T1 cell line following 1 h treatment with 1 µM ouabain, (*p* = 0.7, *n* = 5). Significance was assessed using a two-tailed unpaired t-test, ns *p* > 0.05, * *p* < 0.05, ** *p* < 0.01, *** *p* < 0.001, **** *p* < 0.0001. Data plotted as mean ± SD

All data are presented as mean ± standard deviation (SD). Data acquired during 24 h ouabain exposure MTT (Fig. 1g) were transformed to % of untreated control viability and then an empirical nonlinear fit was applied to determine the EC_50_ for each cell line. Two-tailed unpaired t-tests (assuming equal SD) were used for vehicle versus ouabain-treated samples where *p* < 0.05 was taken to be significant and * denotes *p* < 0.05, ** *p* < 0.01, *** *p* < 0.001, **** *p* < 0.0001. Statistical tests were performed using GraphPad Prism. Unclassified principal components analysis (PCA) was performed in MATLAB. Metabolite peak integrals were first normalised to total spectrum area and was then auto-scaled prior to PCA calculation using Venetian blinds cross validation and five cross validation groups. Hierarchical cluster analysis was performed in MATLAB using the function clustergram.

## Results

### Metabolic profiles

Metabolic profiles were first characterized for the different cells using NMR-based metabolomics. Intracellular metabolite concentrations were measured in the three human cancer cell lines (MDA-MB-231, HCC1954, and MCF-7), in the mouse cancer cells (4T1) and in the control human epithelial cells (MCF-10A). A full table of data is given in Supplementary Table S2, and representative annotated ^1^H spectra are shown in Fig. S2. Principle component analysis, Fig. 1a, and hierarchical cluster analysis, Supplementary Fig. S3, showed a good separation of intracellular metabolites in the different cell lines, highlighting distinct metabolic profiles. Intracellular lactate, Fig. 1b, was significantly higher in all cancer cells with respect to control epithelial cell line, (left panel, mmol (L cell volume)^−1^, *n* = 5): MCF-10A: 4.4 ± 0.4, 4T1: 23.3 ± 2.6 (*p* < 0.0001), MDA-MB-231: 27.3 ± 2.1 (*p* < 0.001), HCC1954: 25.9 ± 4.7 (*p* < 0.01), MCF-7: 34 ± 4 (*p* < 0.001). Intracellular phosphocholine (a marker of phospholipid membrane turnover in proliferating cells), Fig. 1c, was higher in all cancer cells with respect to control epithelial cell line, (right panel, mmol (L cell volume)^−1^, *n* = 5), MCF-10A: 1.05 ± 0.06, 4T1: 10.9 ± 0.5 (*p* < 0.0001), MDA-MB-231: 14.5 ± 0.6 (*p* < 0.0001), HCC1954: 5.4 ± 0.8 (*p* < 0.001), MCF-7: 11.0 ± 1.0 (*p* < 0.0001).

The concentrations of extracellular metabolites in the culture medium of each cell line were measured after 24 h at 37 °C; the ^1^H NMR spectra of respective control media were subtracted to measure metabolite utilisation or excretion. A full table of data is given in Supplementary Table S3, and representative annotated subtracted ^1^H spectra are shown in Fig. S4. Glucose consumption, Fig. 1d, was significantly higher in all cancer cells, compared with the control epithelial cell line, (µmol 10^–6^ cells, *n* = 5): MCF-10A: 0.11 ± 0.03, 4T1: 0.52 ± 0.02, MDA-MB-231: 0.51 ± 0.03, HCC1954: 0.57 ± 0.02, MCF-7: 0.50 ± 0.02. Glutamine utilisation, Fig. 1e, was significantly higher in all cancer cells, compared with the control epithelial cell line, (µmol 10^–6^ cells, *n* = 5): MCF-10A: 0.013 ± 0.002, 4T1: 0.095 ± 0.004, MDA-MB-231: 0.049 ± 0.002, HCC1954: 0.054 ± 0.002, MCF-7: 0.045 ± 0.001. Lactate excretion, Fig. 1f, was significantly higher in all cancer cells, compared with the control epithelial cell line, (µmol 10^–6^ cells, *n* = 5): MCF-10A: 0.15 ± 0.01, 4T1: 0.54 ± 0.05, MDA-MB-231: 0.44 ± 0.02, HCC1954: 0.62 ± 0.01, MCF-7: 0.42 ± 0.02 µmol 10^–6^ cells.

Taken together, these data are indicative of all four breast cancer cell lines displaying significantly elevated aerobic glycolysis compared to the control epithelial cell line.

### Ouabain cytotoxicity – establishing EC_50_

Ouabain is an inhibitor of NKA, with reported effects on cell proliferation and survival [35, 36]. The methylthiazolyl tetrazolium (MTT) cell-cytotoxicity assay was used to determine the concentration of ouabain that led to a defined level of cell death over 24 h, Fig. 1g. Human breast cancer cells showed differing sensitivity to ouabain, with EC_50_ values in the sub micromolar range of 0.5 µM (MDA-MB-231), 0.2 µM (HCC1954), and 0.04 µM (MCF-7). For the murine 4T1 cell line, EC_50_= 40 µM, thus showing that it was 80–1000-fold less sensitive to ouabain, as reported for cells derived from rodents [37–39]. Given the EC_50_ estimates, exposure to 1 µM ouabain for 1 h was chosen for subsequent experiments, which is above the 24 h EC_50_ for the human cells and below the EC_50_ for the 4T1 cells. Since the MTT cytotoxicity assay reports on metabolism as a surrogate measure of viability, the trypan blue exclusion assay was used to measure membrane integrity to assess viability more directly in the absence of acute effects on metabolism. Figure 1h shows that 1-h acute exposure to ouabain did not reveal any cytotoxicity with 100% viability as assessed by trypan blue exclusion.

### ^23^Na NMR spectroscopy of [Na^+^]_i_

Triple quantum filtered (TQF) ^23^Na-NMR is an established method [34] for non-invasively measuring [Na^+^]_i _in red blood cells [40], the perfused heart [31, 41] and other tissues [42]. We adapted the method for cancer cell suspensions using 5-mm OD Shigemi NMR tubes, which retain the cells within the active volume of the radio-frequency (RF) coils of the NMR probe. The method detects Na^+^ that is in exchange-binding to macromolecules, while filtering out the signal from unbound extracellular [Na^+^]_e_, yielding a spectral peak with an intensity that is proportional to [Na^+^]_i_. A reference capillary (inserted into the cell suspension) containing a set agarose gel, with the shift reagent Tm-DOTP, together with a known concentration of NaCl (140 mM), yielded a TQF peak that was shifted away from the intracellular one, Fig. 1i, thus enabling normalization of the relative peak areas. The peak area of the ^23^Na-TQF signal was linear with cell number across all cancer cell lines, Fig. 1i and Supplementary Fig. S5. The ^23^Na-TQF signal was normalised to the internal standard, cell number (10^6^), and cell volume (pL) enabling the measurement of relative changes in [Na^+^]_i_. All cancer cells displayed elevated [Na^+^]_i_ compared with the breast epithelial cell line MCF-10A, Fig. 1j (filled symbols).

Control cell suspensions maintained a stable [Na^+^]_i_ over a 1 h time course, whereas ouabain treatment led to the steady accumulation of [Na^+^]_i_ within the same period, Supplementary Fig. S6. Treatment with 1 µM ouabain for 1 h led to a significant increase in [Na^+^]_i_ in all human cancer cells compared to vehicle control, Fig. 1j (open symbols, *n* = 5), MDA-MB-231 by 188 ± 23% (*p* < 0.0001); HCC1954 by 90 ± 40% (*p* = 0.0101); MCF-7 by 36 ± 12% (*p* = 0.0127). Ouabain elicited no effect on [Na^+^]_i_ in the murine 4T1 cells compared to vehicle control, implying that the increase in the human cells was due to NKA inhibition.

### Treatment effect of ouabain on extracellular acidification rate

**Fig. 2:**
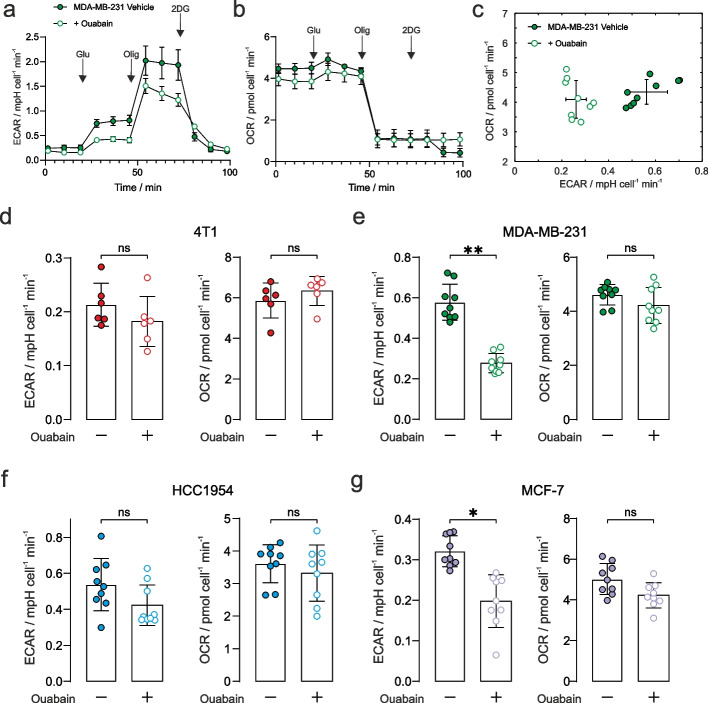
Extracellular acidification rate with NKA inhibition**. a** Seahorse XFe24 glycolytic stress test of extracellular change in pH for the MDA-MB-231 cancer cells (± SEM representing biological reproducibility of *n* = 3 biological repeats where each *n* = 3 technical repeats were first averaged). Complete time courses for the other cell lines are given in Supplementary Fig. S8. The stress test comprised 10 mM glucose, 1 μM oligomycin, and 100 mM 2-deoxy-D-glucose indicated by arrows. **b** Oxygen consumption rate (OCR) measured simultaneously with the ECAR data in panel **a**. **c** Plot of the measured ECAR glycolytic rate vs OCR during the 10 mM glucose time window defined in Supplementary Fig. S6 (*n* = 3 biological repeats each with *n* = 3 technical repeats) showing a reduction in glycolytic rate and no change in OCR. Quantified extracellular acidification rate corresponding to glycolytic rates during the 10 mM glucose time window (left panels) and their corresponding OCR (right panels), in control and ouabain treated cells: **d** 4T1: ECAR (*p* = 0.24), OCR (*p* = 0.39); **e** MDA-MB-231: ECAR decreased by 52% (*p* = 0.006), OCR (*p* = 0.40); **f** HCC1954: ECAR decreased by 21% (*p* = 0.08), OCR (*p* = 0.66); **g** MCF-7: ECAR decreased by 38% (*p* = 0.015), OCR (*p* = 0.16). *n* = 3 biological repeats each with n = 3 technical repeats, significance was assessed using a nested unpaired t-test. ns *p* > 0.05, * *p* < 0.05, ** *p* < 0.01

Seahorse XFe24 (ECAR) flux-analysis used a commercial multi-well instrument and prescribed protocol to study glycolytic flux through to lactic acid (and hence a change in pH; a proxy for glycolytic rate) and oxygen consumption (OCR) in an adherent monolayer of cells. It was applied to the panel of cancer cells that had been pre-treated with 1 µM ouabain for 1 h. Figures 2a and 2b show the ECAR and OCR time courses plotted for the MDA-MB-231 cells, with ECAR vs OCR shown in Fig. 2c, demonstrating significant decreases in ECAR, with no change in OCR during the addition of 10 mM glucose, in the presence of ouabain. Time courses for the other cells are given in Supplementary Fig. S9. Figures 2d-2g show the quantified ECAR values and OCR after the addition of 10 mM glucose. Ouabain elicited a reduction in glycolytic rate in the MDA-MB-231 and MCF-7 cells, while rate-change in HCC1954 cells did not reach significance. Ouabain elicited no effect on ECAR in the murine 4T1 cells showing that decreased glycolytic rate was a consequence of NKA inhibition by ouabain. We recorded no difference in OCR in the four cancer cell lines, indicating that there was no change in oxidative metabolism (Figs. 2d-2g).

### ^2^H NMR of glycolytic flux

**Fig. 3:**
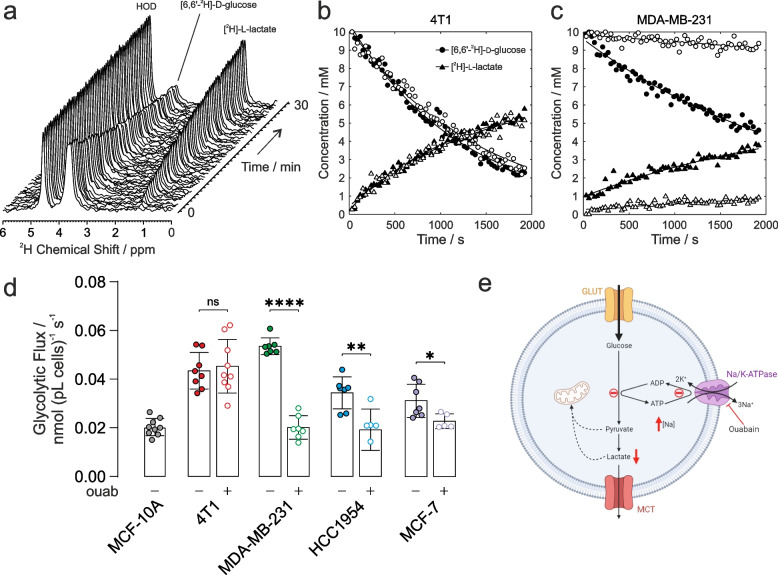
Glycolytic flux measured with ^2^H-NMR. **a** Time-series of ^2^H-NMR spectra showing metabolism of [6,6-^2^H_2_]d-glucose to [3,3-^2^H_2_]l-lactate by 4T1 cells in suspension. No ^2^H label was lost to HOD, serving as an internal chemical shift and intensity standard at 16.7 mM. Time course and empirical fits performed in Matlab of the normalized peak integrals of the [6,6-^2^H_2_]d-glucose and [3,3-^2^H_2_]l-lactate spectral peaks: **b** 4T1 cells and **c** MDA-MB-231 cells for vehicle control (filled symbols) and ouabain treated (open symbols). **d** Quantified glycolytic flux in MCF-10A cells was 0.020 ± 0.003 nmol (pL cells)^−1^ s^−1^ (*n* = 9). 4T1 cells had a higher baseline glycolytic rate of 0.043 ± 0.007 nmol (pL cells)^−1^ s^−1^ (*n* = 8) and unchanged rate after ouabain treatment, 0.045 ± 0.010 nmol (pL cells)^−1^ s^−1^ (*n* = 9, *p* = 0.6949). Human breast cancer cells all showed higher baseline glycolytic rates than control epithelial cells, MDA-MB-231: 0.054 ± 0.003 nmol (pL cells)^−1^ s^−1^ (*n* = 7); HCC1954: 0.034 ± 0.006 nmol (pL cells)^−1^ s^−1^ (*n* = 7); MCF-7: 0.031 ± 0.006 nmol (pL cells)^−1^ s^−1^ (*n* = 7). Human cells showed a decreased glycolytic rate following ouabain-treatment vs vehicle control, MDA-MB-231: 0.020 ± 0.004 nmol (pL cells)^−1^ s^−1^ (*n* = 7; *p* < 0.0001); HCC1954: 0.019 ± 0.008 nmol (pL cells)^−1^ s^−1^ (*n* = 6; *p* = 0.004); MCF-7: 0.023 ± 0.003 nmol (pL cells)^−1^ s^−1^ (*n* = 5; *p* = 0.029). **e** Schematic of the proposed mechanism of the effect of ouabain inhibition of NKA on glycolytic flux (Figure created using BioRender.com). ns *p* > 0.05, * *p* < 0.05, ** *p* < 0.01, **** *p* < 0.0001. Data plotted as mean ± SD

The above glycolytic flux experiments reported on the terminal H^+^ production measured by extracellular acidification rate (ECAR) as a surrogate for actual glycolysis. De Feyter et al. (2018) have shown that metabolism of [6,6-^2^H_2_]d-glucose via glycolysis to [3,3-^2^H_2_]l-lactate (or [3-^2^H]L-lactate) can be followed non-invasively in cells, and in vivo in real time, yielding quantitative estimates of the rate of glucose consumption and lactate production. Accordingly, cancer cells were resuspended in glucose-free Dulbecco’s modified Eagle’s medium (DMEM) that was supplemented with 10 mM [6,6-^2^H]d-glucose then immediately placed in the NMR spectrometer. ^2^H NMR spectra were acquired every 30 s for ~ 30 min to quantify the rate of glucose consumption, and lactate production (Fig. 3a). Importantly, there was no significant loss of ^2^H label to water in the cell suspension; hence, deuterated water (HOD) conveniently served as an internal chemical shift and peak-intensity reference for these studies, [^2^H]HOD has a natural abundance in water of 16.7 mM. Figure 3b shows time courses of the peak integrals of [6,6-^2^H_2_]d-glucose and [3,3-^2^H_2_]l-lactate for 4T1 cells and Fig. 3c for MDA-MB-231 cells; vehicle treated (filled symbols) and 1µM ouabain treated (open symbols). Decreased glycolytic flux can be seen in MDA-MB-231 cells following ouabain treatment, while no change in rate is seen in the 4T1 cells.

Figure 3d shows the calibrated rates of glycolytic flux derived from fitting the data in MATLAB, normalised to cell number (10^6^) and cell volume (pL). All cancer cells showed significantly higher baseline glycolytic rates than the healthy epithelial cell line, giving further evidence of the Warburg phenotype in the chosen panel of cancer cells. Glycolytic rates measured in the human breast cancer cells were all significantly lower following ouabain treatment vs vehicle controls, while the glycolytic rate measured in the murine 4T1 cells was unchanged following ouabain treatment.

### Membrane permeabilization with Gramicidin-A

To investigate whether there was a graded relationship between [Na^+^]_i_, NKA activity, and glycolytic rate, we used gramicidin-A to permeabilise the plasma membranes to monovalent cations to allow the intracellular [Na^+^]_i_ concentration to be matched to a series of extracellular concentrations. Extracellular solutions were designed to be isotonic with Na^+^ replaced with K^+^ to give a range of NKA-activating Na^+^ concentrations, while [Ca^2+^] and pH were held constant (see Supplementary Table S1). NKA is maximally activated at all K^+^ concentrations above 10 mM and hence extracellular [K^+^] did not limit the rate under these conditions.

**Fig. 4:**
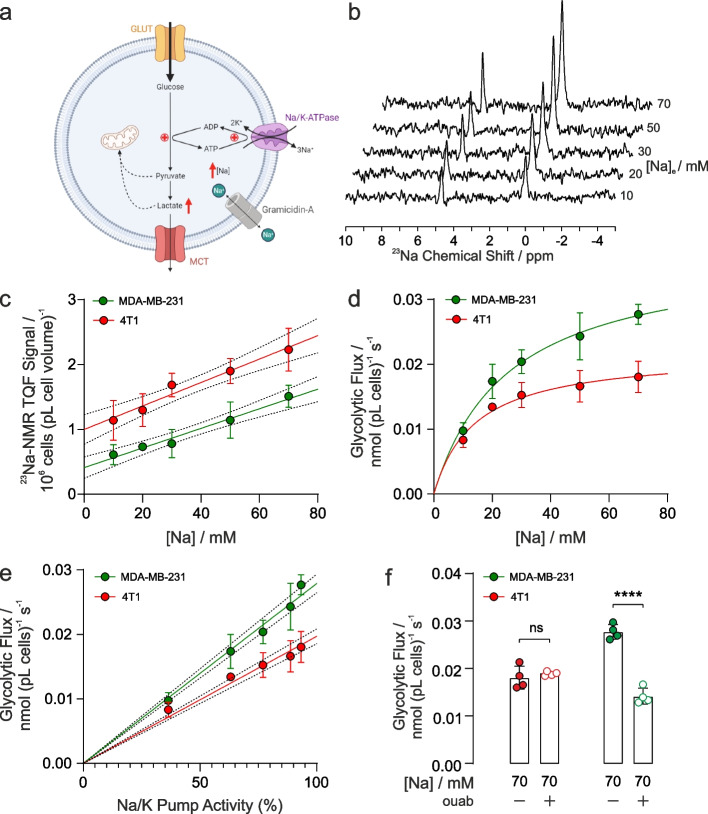
Glycolytic flux affected by intracellular [Na]_i_ and NKA function. **a** Schematic of the proposed mechanism of the effect of gramicidin-A on [Na^+^]_i_ and glycolysis. Gramicidin introduces an artificial Na^+^ leak, increasing [Na^+^]_i_ and glycolytic metabolism (Figure created using BioRender.com). **b** ^23^Na TQF spectra showing the intracellular [Na^+^]_i_ peak relative to a reference capillary in MDA-MB-231 cells following membrane permeabilization with gramicidin and varying concentrations of titrated extracellular [Na^+^]_e_. **c** Quantification of TQF ^23^Na NMR spectra (proportional to [Na^+^]_i_) following exposure to isotonic solutions of titrated [Na^+^]_e_ (*n* = 4), for 4T1 cells and MDA-MB-231 cells. **d** Quantification of glycolytic fluxes measured by the rate of [6,6-^2^H_2_]d-glucose to [3,3-^2^H_2_]l-lactate conversion at different concentrations of titrated [Na^+^]_i_ in murine 4T1 and human MDA-MB-231 breast cancer cells (*n* = 4). **e** Glycolytic fluxes in panel **d** replotted as a function of pump current derived from the analytical expression given by Silverman et al. [43] **f** Glycolytic flux measured at the highest concentration of 70 mM [Na^+^]_e_ following treatment with 1 µM ouabain treatment was not significantly altered in murine 4T1 cells but was significantly decreased in human MDA-MB-231 cells. ns *p* > 0.05, **** *p* < 0.0001. Data plotted as mean ± SD

Upon permeabilization with gramicidin-A, and resuspension of the cells in buffers with [Na^+^]_e_ ranging from 10 – 70 mM, intracellular [Na^+^]_i_ (measured by ^23^Na TQF NMR) showed a linear dependence on [Na^+^], Fig. 4b and 4c, in both the murine 4T1 and the human MDA-MB-231 breast cancer cells. Under the same titrated conditions of [Na^+^] ranging from 10 – 70 mM, the rates of glycolytic flux measured by ^2^H-NMR correlated directly with increasing [Na^+^]_i_ in both cell lines, Fig. 4d. The K_m_ for Na^+^ from the plots in Fig. 4d were 14 mM for 4T1 cells and 25 mM for MDA-MB-231 cells with a similar V_max_ (plateau) at high [Na^+^]. NKA pump current (activity) has been previously measured as a function of [Na^+^] using patch clamping and an analytical expression has been reported (Fig. 3 in Silverman et al. [43]). Using this expression and converting [Na^+^] into NKA pump current (as a % of maximum), glycolytic flux was found to be directly proportional to pump current (NKA activity), Fig. 4e. Finally, Fig. 4f shows the glycolytic fluxes measured at the highest [Na^+^]_i_ = 70 mM (closed symbols) and when treated with ouabain (open symbols), which was unchanged in 4T1 cells whereas there was nearly a 60% decrease in glycolytic flux in ouabain treated MDA-MB-231 cells.

### In vivo PET imaging of tumour metabolism

**Fig. 5:**
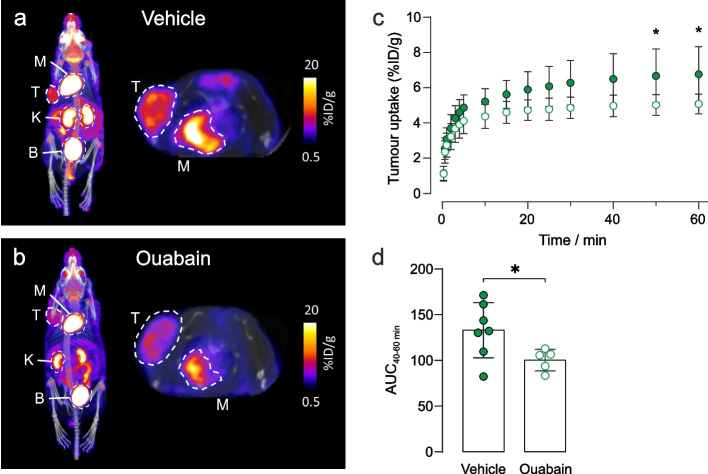
Ouabain decreases ^18^F-FDG uptake in MDA-MB-231 tumour xenografts. Representative images of the biodistribution of ^18^F-FDG in **a** vehicle and **b** ouabain treated tumours acquired at 60 min scan time. The tumour and organs of interest are denoted by dotted circles, where T = tumour; K = kidney, M = myocardium, B = bladder. **c** The pharmacokinetics of ^18^F-FDG uptake were determined via the time-activity curves. A decreased avidity for ^18^F-FDG was observed in tumours following treatment with ouabain (6.75 µM final blood concentration). At 50 min post ^18^F-FDG injection, uptake was significantly lower in ouabain treated tumours 5.0 ± 0.6%ID/g (*n* = 5) compared to control tumours 6.7 ± 1.6%ID/g (*n* = 7), *p < 0.05, two-way ANOVA with Šidák’s multiple comparisons. **d** The area under the curve (AUC) derived from the time-activity curves at 40–60 min post injection of ^18^F-FDG revealed a significant decrease in the uptake of ^18^F-FDG in ouabain treated, AUC_40-60 min_ = 100 ± 12 (*n* = 5) versus vehicle controls, AUC_40-60 min_ = 130 ± 40 (*n* = 7), *p < 0.05, two-tailed unpaired t-test. Data plotted as mean ± SD

To investigate tumour metabolism in vivo, we used ^18^F-FDG, an analogue of glucose that is used in clinical oncology to assess tumour burden and treatment response. Immunodeficient mice bearing subcutaneous human MDA-MB-231 xenograft tumours were injected intravenously with vehicle ± ouabain 1 h prior to injection with ^18^F-FDG. At 20 min post injection (p.i.) of ^18^F-FDG, mice were scanned for 1 h and PET data were reconstructed dynamically to evaluate pharmacokinetics and biodistribution. Representative images demonstrated decreased tumour uptake of ^18^F-FDG in ouabain-treated compared to vehicle controls (Figs. 5a and 5b). The difference in uptake was significant from 40–60 min scan time (Figs. 5c) in-line with clinical protocols, which add a 1 h delay between injection and PET scanning [44]. The area under the curve of 40–60 min scan time revealed a significant decrease in ^18^F-FDG uptake of -24.5% in ouabain-treated versus vehicle treated controls, Fig. 5d. The biodistribution (major organ uptake) of ^18^F-FDG was comparable between groups in all other murine tissues, demonstrating the insensitivity of murine cells to cardiotonic steroids compared to human cells (Supplementary Fig. S11).

## Discussion

### Intracellular [Na^+^]_i_—correlation with glycolytic flux

Our experiments demonstrated a direct, functional link between intracellular [Na^+^]_i_, NKA activity, Na^+^ membrane permeability, and glycolytic flux in the breast cancer cells. Acute inhibition of NKA with 1 µM ouabain in the human cells MDA-MB-231, HCC1954 and MCF-7 resulted in significantly elevated [Na^+^]_i_ and a concomitant decrease in glycolytic flux. Treatment of murine 4T1 cells with 1 µM ouabain did not alter [Na^+^]_i_ nor glycolytic flux, due the lack of inhibition, and therefore acted as a negative control. Murine cells are typically less sensitive to ouabain than human cells due to two amino acid substitutions in the K^+^binding site of the α1 subunit of NKA (Q111R, N122D) causing ~ 1000-fold decreased ouabain sensitivity [37–39]. These experiments demonstrated the direct association between NKA activity and the ‘energy demand regulation’ of glycolytic flux. A schematic of the proposed mechanism is shown in Fig. 3e.

The influence of membrane permeability of Na^+^ on glycolytic flux was demonstrated in human MDA-MB-231 cells, and in murine 4T1 cells by using gramicidin-A. Gramicidin inserts into the plasma membrane (and has also been reported to insert into the mitochondrial membrane) creating a mono-valent cation-permeable pore. Under these conditions, the Na^+^ (and K^+^ and H^+^) concentrations equilibrate (become equal) across the membrane(s) removing any trans-membrane Na^+^ gradient and dissipating the membrane potential(s). Dissipating the mitochondrial H^+^ gradient will inhibit mitochondrial ATP synthesis and therefore metabolism is expected to be dominated by glycolysis. Using modified extracellular buffers where [Na^+^]_i_ was tightly controlled, ranging from 10 – 70 mM, the ^23^Na TQF experiments showed a linear dependence on [Na^+^]. Our ^23^Na TQF experiment preferentially report on intracellular Na^+^, however, due to the mechanism of triple quantum filtering, which selects for Na^+^ that is slowly tumbling due to its association with larger macromolecules, there is a contribution to the signal both from intracellular Na^+^ bound to protein and a contribution from extracellular Na^+^ bound to the surfaces of membranes. This gives an offset to the TQF signal. Thus, while the TQF signal is proportional to [Na^+^]_i_ it is not directly so, ie. a calibration line of signal intensity versus [Na^+^] does not pass through the origin.

Enhanced glycolytic flux correlated directly with increasing [Na^+^]_i_ in both cell lines, which could subsequently be inhibited with 1 µM ouabain in MDA-MB-231 cells, but not in the 4T1 cells. The K_m_ values found from the fits in Fig. 4d are close to the reported K_m_ value of 15.5 mM for NKA pump current (activity) and [Na^+^] [43]. The Na^+^ dependence of glycolysis is therefore dependent on the kinetics of NKA. Glycolytic fluxes, Fig. 4d, showed a plateau (V_max_) at high [Na^+^] which could reflect the saturation of NKA by Na^+^ or the saturation of glycolysis and glucose transport. When [Na^+^] is converted to pump activity using the expression of Silverman et al. [43] glycolytic fluxes show a linear dependence on activity. Hence, the plateaux in the plots of glycolytic flux in Fig. 4d are due to saturation of NKA by Na^+^ at its V_max_ and not due to saturation of glycolytic flux or glucose transport. This suggests that over the range of [Na^+^] that we used, glucose supply and glycolysis were not rate determining for the NKA. Thus, what limits glycolytic flux at high [Na^+^] concentration in Fig. 4d is the saturation of NKA by Na^+^ and not [ATP] supply. A further interesting point in the curve fits of glycolytic activity vs [Na^+^], Fig. 4d, is that they pass through the origin when it is extrapolated to [Na^+^] = 0 mM. This finding suggests that in the presence of gramicidin, when the [Na^+^], [K^+^] and [H^+^] gradients across the membrane are dissipated (and when [Ca^2+^] is low there will very little residual Ca-ATPase activity), glycolytic activity would be very low (or zero). Of relevance to this interpretation, the magnitudes of glycolytic flux measured with gramicidin-A present were lower than those measured in unpermeabilised cells. It is possible that treatment with gramicidin-A led to a reduction in other related ATPase activities, notably H^+^-ATPases due to dissipation of the H^+^ gradient. Given these observations/ deductions it is notable that ouabain did not completely inhibit glycolytic flux in the MDA-MB-231 cells; this probably resulted from using a submaximal concentration of ouabain. These experiments demonstrated that [Na^+^]_i_ directly contributes to the enhanced glycolytic flux observed, and that this was dependent on NKA activity. In other words, the [Na^+^]_i_ dependent increase in NKA activity caused increased energy demand (ATP turnover) of the pump that led to stimulation of ATP supply by glycolysis, thus matching this demand. A schematic of the proposed mechanism is shown in Fig. 4a.

Elevated basal [Na^+^]_i_ in cancer cells is predicted to push NKA up its activation curve leading to an enhancement of glycolytic flux. Taken together, our data can be understood in the light of the known negative feedback controls of ATP/ADP cycling and glycolysis in which accumulation of ATP allosterically inhibits hexokinase, and MgATP of PFK-1 [45]; these reactions that have high flux control coefficients in glycolysis [46]. Therefore, inhibition of NKA leads to a significant reduction in overall ATP turnover by the cell, causing almost immediate reductions in glycolytic flux. Elevated [Na^+^]_i_ (whether due to increased influx or decreased efflux) also has the potential to modulate intracellular pH through the action of NHE and CA-NBC. The H^+^gradient across the plasma membrane is known to be reversed in cancer cells with acidification of the extracellular environment and alkalinisation of the cytosol. This also drives accelerated glycolysis, while acidification of the intracellular environment is known to inhibit glycolysis [47]. Since the H^+^ gradient is abolished by gramicidin-A in our experiments then intracellular acidification would not contribute to the reduced rates of glycolysis observed in MDA-MB-231 cells on ouabain treatment.

### The Warburg effect

In the ~ 100 years since the discovery by Otto Warburg of the upregulation of glycolysis in cancer cells [3], there have been multiple hypotheses that “explain” the basis of this phenomenon. Most recent hypotheses posit genome, transcriptome, and proteomic-level alterations in cancer cells that underlie the uncontrolled cell proliferation, which is the hallmark of neoplasia [48]. There is strong evidence that ‘metabolic reprogramming’ of cancer cells leads to an advantageous phenotype with greater anabolic capacity of branch pathways; this occurs via upregulation of glycolysis, the PPP, decreased TCA cycle activity, as well as modifications of the extracellular environment (e.g., lower pH), leading to modification of the extracellular matrix, immune cell evasion, and genetic reprogramming thus favouring natural selection via survival of the fittest cells [49].

Previous studies have reported a link between the NKA and glycolysis in several cell types: erythrocytes [17], neuronal [16], skeletal [50, 51], and cardiomyocytes [19]. Our other recent work further demonstrates the importance of the NKA and elevated intracellular [Na^+^]_i_ as a modulator of cardiac metabolism [52] with a switch in metabolism away from fatty acid oxidation towards glucose utilisation in ouabain-treated hearts, with similar observations in hypertrophic hearts and transgenic phospholemman (PLM^3SA^) mice, that have chronic cardiac myocyte [Na^+^]_i_elevation [52]. Paradoxically, the metabolic response that we observed to ouabain in cancer cells is *opposite *to what we observed in the beating heart in which ouabain is known to increase cardiac contractility and substrate consumption [52].

Numerous studies have reported upregulated expression of voltage gated sodium channels (VGSCs) and other Na^+^-ion transporters in several cancers [53–59]. Further work supports the anti-cancer properties of Na^+^-channel ‘blockers’ [60] as well as NKA inhibitors [61]. There is mounting evidence as to how these changes in ion channel expression or kinetics provide a selective advantage for cancer cell survival (and perhaps morphogenesis) [25, 62]. The data presented here, indicate that alterations to Na^+^ ion homeostasis across the plasma membrane can directly affect glycolytic rate in breast cancer cells. Since glycolysis and ATPase activity both generate H^+^, elevated proton efflux facilitates extracellular acidosis which provides tumours with one aspect of their ‘naturally selective’ microenvironment. However, intracellular pH will also play an important role in this mechanism since H^+^ efflux via NHEs would further amplify the Na^+^ leak into the cell.

Our hypothesis is of relevance in cancer imaging, underpinning the practice of clinical ^18^F-FDG PET. Specifically, we demonstrated that ouabain treatment of a murine human MDA-MB-231 xenograft model led to decreased tumour uptake of ^18^F-FDG, while murine tissues were unaffected thus further evidencing NKA-dependency of glucose demand in tumours. Indications from clinical ^23^Na-MRI also support the finding that tumours have elevated [Na^+^]_i _compared with surrounding healthy tissue [22, 23], while ^2^H-MRI and ^82^Rb-PET imaging are emerging techniques in clinical oncology [63–66].

A further consequence of altered Na^+^influx in cells is the impact that this has on the maintenance of a depolarized membrane [67]. Cone et al. demonstrated that mitotic cell division and proliferation rates are controlled by membrane depolarization [68–72]. Thus, an increased Na^+^permeability could both increase NKA-driven glycolysis, and hence metabolite availability, while also promoting rapid cell proliferation. Although the current study has not identified the structural proteins and enzymes that are most likely to lead to the functional juxtaposition of glycolysis with the NKA, we have identified a major sink for the glycolytic ATP production in a manner that is similar to what occurs in the human erythrocyte [17]. It is possible that this functional proximity is indeed a structural one where key glycolytic enzymes are associated with the plasma membrane [11], namely a ‘metabolon’. The exact identity of the Na^+^ influx pathways is complex, but it includes the voltage gated Na^+^-channels as well as Na^+^/H^+^ exchangers, NBC, Na^+^K^+^2Cl^−^ cotransporters and others.

## Conclusion

The underlying causes of the Warburg effect observed in cancer cells has been a long-standing conundrum to which the answer is multifaceted. The functional ‘redirection’ of metabolism yields metabolic intermediates that enhance cell growth, and via efflux of lactic acid in particular, a reduction in extracellular pH leading to a modification of the extracellular environment, thus altering the ability to selectively invade surrounding tissue. We demonstrated, further to previous hypotheses, that glycolysis is dependent on ionic homeostasis across the plasma membrane, specifically an Na^+^-dependent consumption of ATP via the NKA. We hypothesise that this is caused by increased Na^+^-influx (which we invoked here with the ionophore gramicidin-A) resulting in increased NKA activity that re-established a steady state of [Na^+^]_i_ that was elevated.

### Supplementary Information

Supplementary Material 1.

## Data Availability

Data are provided within the manuscript or supplementary information files.
