# Supplementary material for: Disrupting Na+ ion homeostasis and Na+/K+ ATPase activity in breast cancer cells directly modulates glycolysis in vitro and in vivo
Source: Cancer Metab. 2024 May 24;12:15. doi: 10.1186/s40170-024-00343-5 (PMC11119389; doi:10.1186/s40170-024-00343-5)
Supplement: Supplementary file 1 — Supplementary Material 1. [file 40170_2024_343_MOESM1_ESM.docx]

**Supplementary Material**

**Disrupting Na^+^ ion homeostasis and Na^+^/K^+^ ATPase activity in breast cancer cells directly modulates glycolysis in vitro and in vivo.**

Aidan M. Michaels,^1^ Anna Zoccarato,^2^ Zoe Hoare,^2^ George Firth,^1^ Yu Jin Chung,^2^ Philip W. Kuchel,^3^ Ajay M. Shah,^2^ Michael J. Shattock,^2^ Richard Southworth,^1^ Thomas R. Eykyn^1*^

^1^ School of Biomedical Engineering and Imaging Sciences, King’s College London, London SE1 7EH, United Kingdom.

^2^ School of Cardiovascular and Metabolic Medicine and Sciences, King’s College London, United Kingdom.

^3^ School of Life and Environmental Sciences, University of Sydney, Sydney, NSW 2006, Australia.

* Address for correspondence: Email: thomas.eykyn@kcl.ac.uk

School of Biomedical Engineering and Imaging Sciences, King's College London, St Thomas' Hospital, London SE1 7EH, United Kingdom


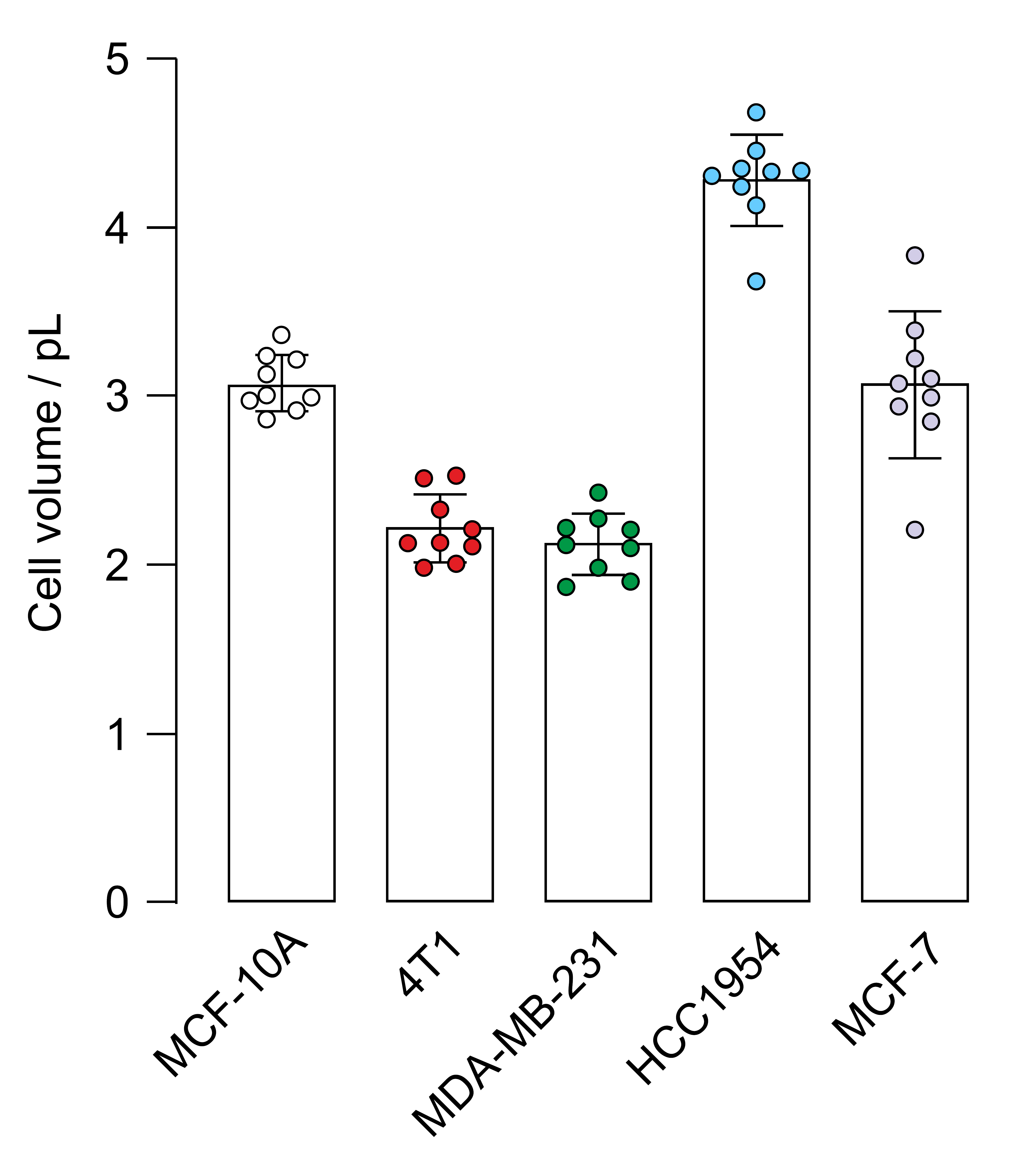


**Supplementary Figure S1. Cell volume measurements.** Cell volume measurements were recorded as regular practice during cell culture passage. Samples were resuspended in serum-free DMEM at a concentration of ~1 x10^5^ cells mL^-1^. MCF-10A = 3.07 ± 0.17 pL, 4T1 = 2.21 ± 0.20 pL, MDA-MB-231 = 2.12 ± 0.18 pL, HCC1954 = 4.27 ± 0.27 pL; MCF-7 = 3.06 ± 0.44 pL. Data is displayed as mean ± SD (n = 9).

**Table S1. NKA activating solutions.** Sodium ions were varied in these solutions and replaced with potassium ions to maintain isotonicity (pH 7.4). EGTA (ethylene glycol-bis(β-aminoethyl ether)-N,N,N′,N′-tetraacetic acid) was added to chelate free Ca to an estimated unbound Ca concentration of 10 µM (calculated using:

<https://somapp.ucdmc.ucdavis.edu/pharmacology/bers/maxchelator/CaEGTA-TS.htm>.

| **Titrated Na buffer (mM)** | | | | | |
| --- | --- | --- | --- | --- | --- |
| NaCl | 10 | 20 | 30 | 50 | 70 |
| KCl | 120 | 110 | 100 | 80 | 60 |
| KOH | 10 | 10 | 10 | 10 | 10 |
| MgCl_2_ | 1 | 1 | 1 | 1 | 1 |
| CaCl_2_ | 1 | 1 | 1 | 1 | 1 |
| EGTA | 1 | 1 | 1 | 1 | 1 |
| Glucose | 10 | 10 | 10 | 10 | 10 |
| L-Glutamine | 4 | 4 | 4 | 4 | 4 |
| HEPES | 10 | 10 | 10 | 10 | 10 |

0.1 mM Gramicidin-A was added to each buffer as a separate bolus before NMR acquisition.

^23^Na NMR acquisitions were performed with unlabelled glucose which was substituted for 10 mM [6,6’-^2^H]-D-glucose-d_2_ in the ^2^H NMR experiments.


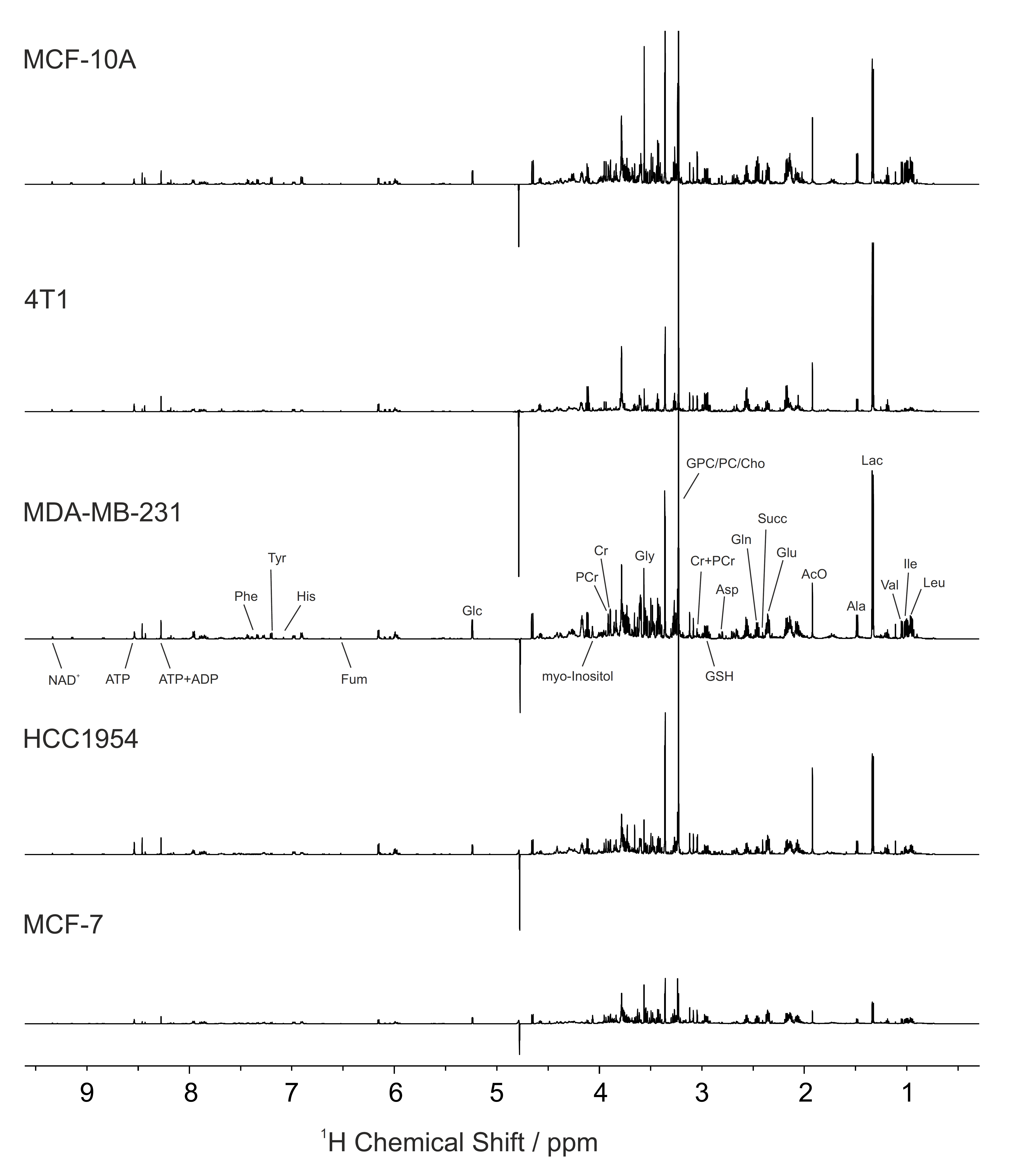


**Supplementary Figure S2. Representative ^1^H NMR spectra of extracted cell metabolites.** Quantified metabolites are annotated in the MDA-MB-231 spectrum.

**Table S2: ^1^H NMR intracellular metabolite concentrations**

|  | **MCF-10A (*n*=5) / mM** | **4T1**  **(*n*=5) / mM** | **MDA-MB-231**  **(*n*=5) / mM** | **HCC1954**  **(*n*=5) / mM** | **MCF-7**  **(*n*=5) / mM** |
| --- | --- | --- | --- | --- | --- |
| NAD | 0.32 ± 0.02 | 0.75 ± 0.06 | 1.01 ± 0.06 | 1.16 ± 0.19 | 1.66 ± 0.19 |
| ATP + ADP | 2.23 ± 0.13 | 7.8 ± 0.5 | 4.30 ± 0.18 | 3.8 ± 0.6 | 4.1 ± 0.5 |
| Formate | 0.37 ± 0.05 | 1.9 ± 0.6 | 3.9 ± 0.6 | 0.38 ± 0.07 | 2.2 ± 0.4 |
| ATP + ADP + AMP | 1.81 ± 0.10 | 6.2 ± 0.4 | 4.19 ± 0.23 | 3.4 ± 0.6 | 4.2 ± 0.6 |
| Phenylalanine | 0.63 ± 0.05 | 0.81 ± 0.07 | 2.01 ± 0.09 | 0.33 ± 0.05 | 2.85 ± 0.29 |
| Tyrosine | 0.57 ± 0.04 | 0.52 ± 0.04 | 1.97 ± 0.09 | 0.23 ± 0.04 | 3.1 ± 0.4 |
| Histidine | 0.21 ± 0.02 | 0.25 ± 0.02 | 0.59 ± 0.02 | 0.12 ± 0.03 | 0.92 ± 0.11 |
| Fumarate | 0.02 ± 0.01 | 0.20 ± 0.02 | 0.12 ± 0.01 | 0.08 ± 0.01 | 0.08 ± 0.01 |
| α-glucose | 8.8 ± 0.8 | 14.1 ± 1.8 | 28.5 ± 1.6 | 2.7 ± 0.6 | 22 ± 4 |
| Myo inositol | 5.2 ± 0.4 | 3.93 ± 0.24 | 7.1 ± 0.4 | 2.6 ± 0.4 | 3.4 ± 0.4 |
| Phosphocreatine | 2.26 ± 0.15 | 4.06 ± 0.21 | 3.34 ± 0.16 | 2.2 ± 0.4 | 7.3 ± 0.8 |
| Creatine | 1.65 ± 0.12 | 4.25 ± 0.20 | 2.47 ± 0.09 | 2.2 ± 0.4 | 6.0 ± 0.6 |
| Glycine | 4.34 ± 0.30 | 5.0 ± 0.4 | 7.4 ± 0.4 | 2.6 ± 0.4 | 16.4 ± 1.9 |
| Glycerophosphocholine | 1.75 ± 0.13 | 2.60 ± 0.14 | 2.30 ± 0.12 | 0.52 ± 0.09 | 4.8 ± 0.7 |
| Phosphocholine | 1.05 ± 0.06 | 10.9 ± 0.5 | 14.5 ± 0.7 | 5.44 ± 0.8 | 11.0 ± 1.0 |
| Choline | 0.22 ± 0.02 | 0.25 ± 0.02 | 0.30 ± 0.02 | 0.11 ± 0.02 | 0.28 ± 0.03 |
| Total creatine | 2.31 ± 0.15 | 4.95 ± 0.25 | 2.35 ± 0.10 | 2.8 ± 0.5 | 7.0 ± 0.8 |
| Glutathione | 9.2 ± 0.6 | 11.6 ± 0.6 | 10.8 ± 0.4 | 12.9 ± 2.0 | 15.3 ± 1.6 |
| Aspartate | 2.66 ± 0.29 | 5.27 ± 0.25 | 6.33 ± 0.24 | 2.5 ± 0.4 | 9.3 ± 1.1 |
| Glutamine | 5.5 ± 0.4 | 6.9 ± 0.4 | 9.20 ± 0.28 | 4.3 ± 0.7 | 21.3 ± 2.3 |
| Succinate | 0.25 ± 0.02 | 1.15 ± 0.06 | 0.51 ± 0.02 | 0.32 ± 0.06 | 0.91 ± 0.08 |
| Glutamate | 10.2 ± 0.7 | 20.6 ± 1.2 | 16.6 ± 0.6 | 6.7 ± 1.1 | 19.2 ± 2.1 |
| Acetate | 1.12 ± 0.11 | 5.6 ± 0.8 | 5.1 ± 0.6 | 3.4 ± 0.7 | 5.6 ± 0.7 |
| Alanine | 1.03 ± 0.07 | 3.34 ± 0.26 | 4.2 ± 0.4 | 2.3 ± 0.4 | 7.14 ± 0.9 |
| Lactate | 4.4 ± 0.4 | 23.3 ± 2.7 | 27.3 ± 2.1 | 26 ± 5 | 34 ± 4 |
| Valine | 0.45 ± 0.03 | 0.40 ± 0.03 | 1.48 ± 0.06 | 0.18 ± 0.03 | 2.16 ± 0.21 |
| Isoleucine | 1.07 ± 0.10 | 1.91 ± 0.10 | 3.33 ± 0.12 | 0.65 ± 0.10 | 4.1 ± 0.4 |
| Leucine | 1.16 ± 0.09 | 2.03 ± 0.13 | 3.76 ± 0.13 | 0.76 ± 0.12 | 4.8 ± 0.5 |

Metabolite concentrations calculated with respect to the TSP internal reference standard and normalized to cell number and cell volume, reported as mean ± standard error in mmol (L cell volume)^-1^.


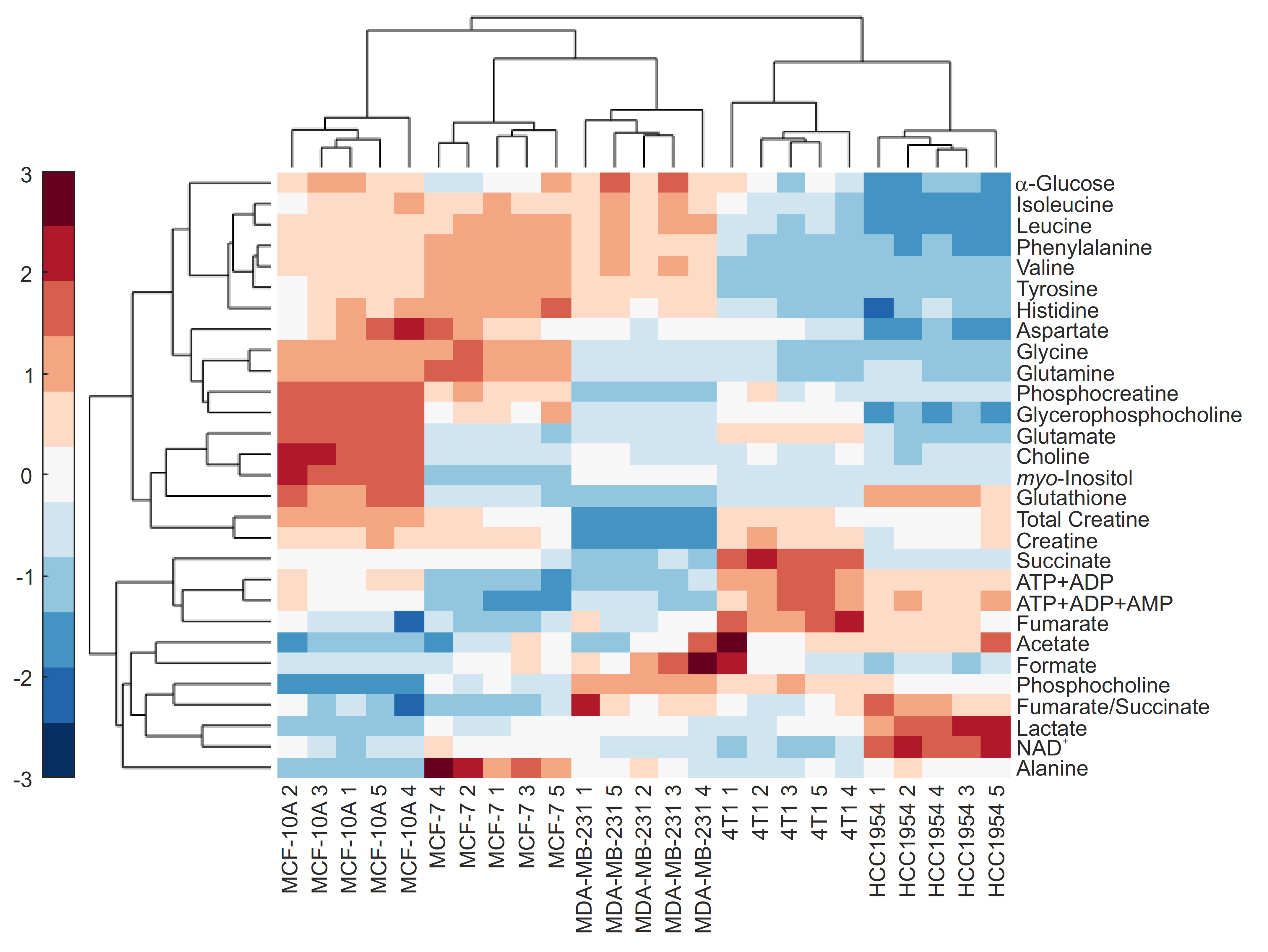


**Supplementary Figure S3. Hierarchical cluster analysis of intracellular metabolites.** The raw data in Table S1 were first normalised to total spectrum integral. Heat maps were calculated in Matlab using the function clustergram. Separate clustering of each individual cell line is seen highlighting different metabolic phenotypes.


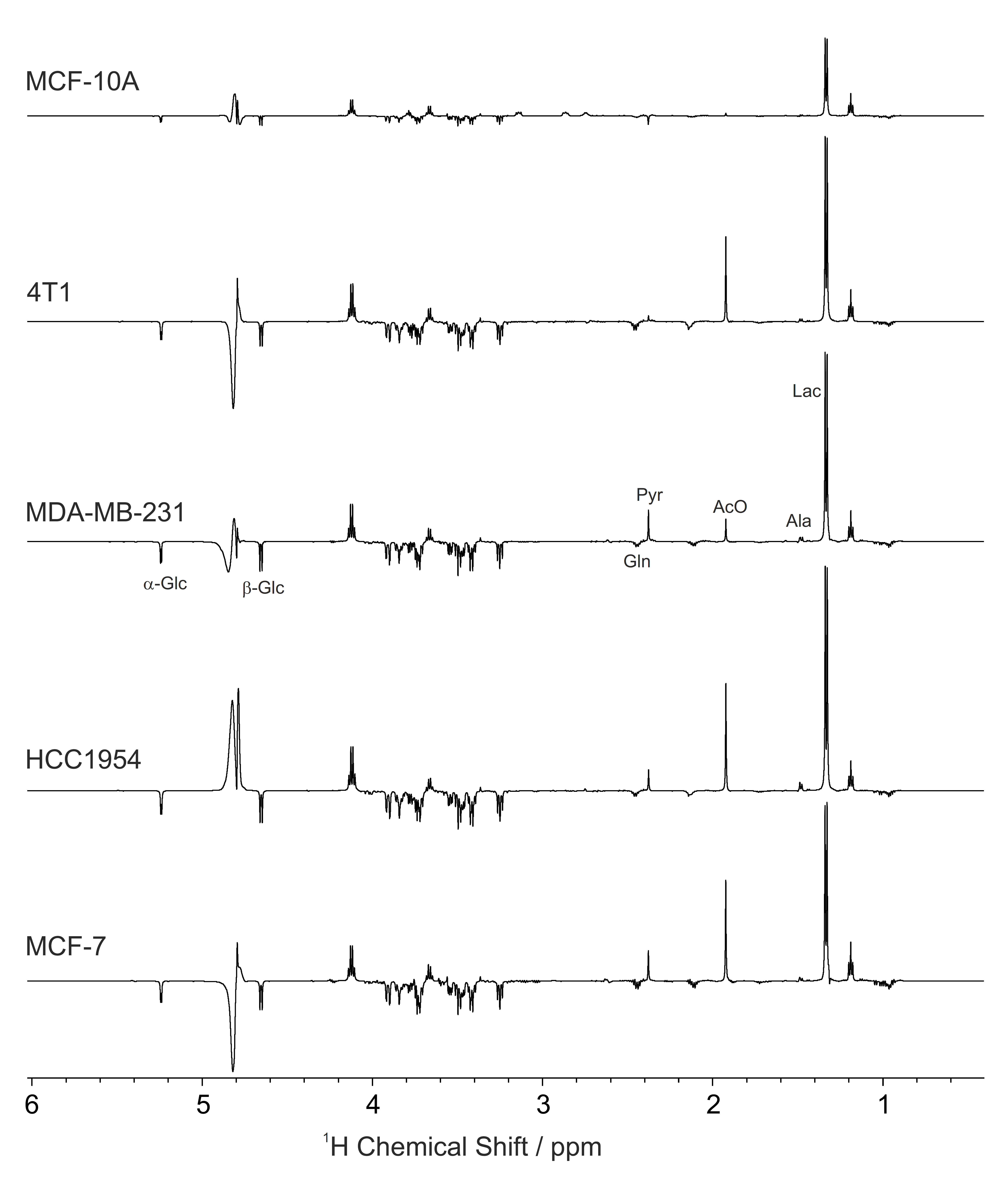


**Supplementary Figure S4. ^1^H NMR spectra of extracellular metabolites in the media.** Difference spectra were calculated by subtracting the respective control fresh medium spectrum from the 24 h cell medium spectrum. Negative peaks correspond to metabolites that were being used by the cells while positive peaks correspond to metabolites excreted from the cells. Quantified metabolites are annotated in the MDA-MB-231 spectrum.

**Table S3: ^1^H NMR metabolite concentrations in the medium after 24 h culture.**

|  | **MCF-10A (*n*=5) / nmol 10^-6^ cells** | **4T1**  **(*n*=5) / nmol 10^-6^ cells** | **MDA-MB-231**  **(*n*=5) / nmol 10^-6^ cells** | **HCC1954**  **(*n*=5) / nmol**  **10^-6^ cells** | **MCF-7**  **(*n*=5) / nmol 10^-6^ cells** |
| --- | --- | --- | --- | --- | --- |
| Formate | 2.1 ± 0.1 | 3.3 ± 0.3 | 1.4 ± 0.1 | 4.8 ± 0.1 | 4.0 ± 0.1 |
| Phenylalanine | -0.3 ± 0.5 | -4.5 ± 0.6 | -3.6 ± 0.5 | -3.1 ± 0.3 | -2.8 ± 0.3 |
| Tyrosine | -0.7 ± 0.3 | -4.0 ± 0.3 | -3.9 ± 0.4 | -3.2 ± 0.2 | -3.6 ± 0.2 |
| Histidine | -0.4 ± 0.2 | -2.4 ± 0.3 | -1.8 ± 0.1 | -2.3 ± 0.4 | -1.6 ± 0.1 |
| α-glucose | -106 ± 25 | -518 ± 21 | -510 ± 30 | -579 ± 18 | -496 ± 18 |
| Aspartate | 1.1 ± 0.3 | -1.3 ± 2.0 | 2.2 ± 0.3 | -11.2 ± 0.7 | 1.3 ± 0.2 |
| Glutamine | -13.2 ± 2.2 | -94.8 ± 4.1 | -49.0 ± 1.5 | -53.6 ± 2.1 | -44.7 ± 1.3 |
| Pyruvate | -6.4 ± 0.1 | 6.2 ± 0.4 | 28.1 ± 0.8 | 16.5 ± 1.0 | 26.0 ± 0.9 |
| Glutamate | 4.3 ± 0.5 | 11.5 ± 1.8 | 14.1 ± 0.3 | -2.9 ± 1.6 | 4.5 ± 0.3 |
| Acetate | 2.3 ± 0.2 | 130 ± 7 | 26.6 ± 0.6 | 161 ± 8 | 114 ± 4 |
| Alanine | 0.7 ± 0.3 | 6.8 ± 1.1 | 12.0 ± 0.6 | 22.5 ± 0.7 | 8.2 ± 0.6 |
| Lactate | 145 ± 6 | 540 ± 50 | 443 ± 20 | 620 ± 10 | 420 ± 21 |
| Valine | -0.9 ± 0.3 | -3.5 ± 0.2 | -4.8 ± 0.4 | -2.7 ± 0.1 | -4.8 ± 0.3 |
| Isoleucine | -3.3 ± 0.5 | -10.0 ± 1.0 | -12.4 ± 0.8 | -11.0 ± 0.4 | -14.8 ± 0.6 |
| Leucine | -4.2 ± 0.6 | -13.1 ± 1.1 | -15.9 ± 1.4 | -13.1 ± 0.6 | -19.7 ± 0.9 |

Metabolite concentrations in 24 h culture medium samples calculated with respect to the TSP internal reference standard. The respective control fresh medium concentrations were subtracted from the 24 h medium samples. Negative values refer to metabolite consumption while positive values refer to metabolite excretion by the cells. Number of moles of each metabolite were normalized to cell number and reported as mean ± standard error in nmol 10^-6^ cells.


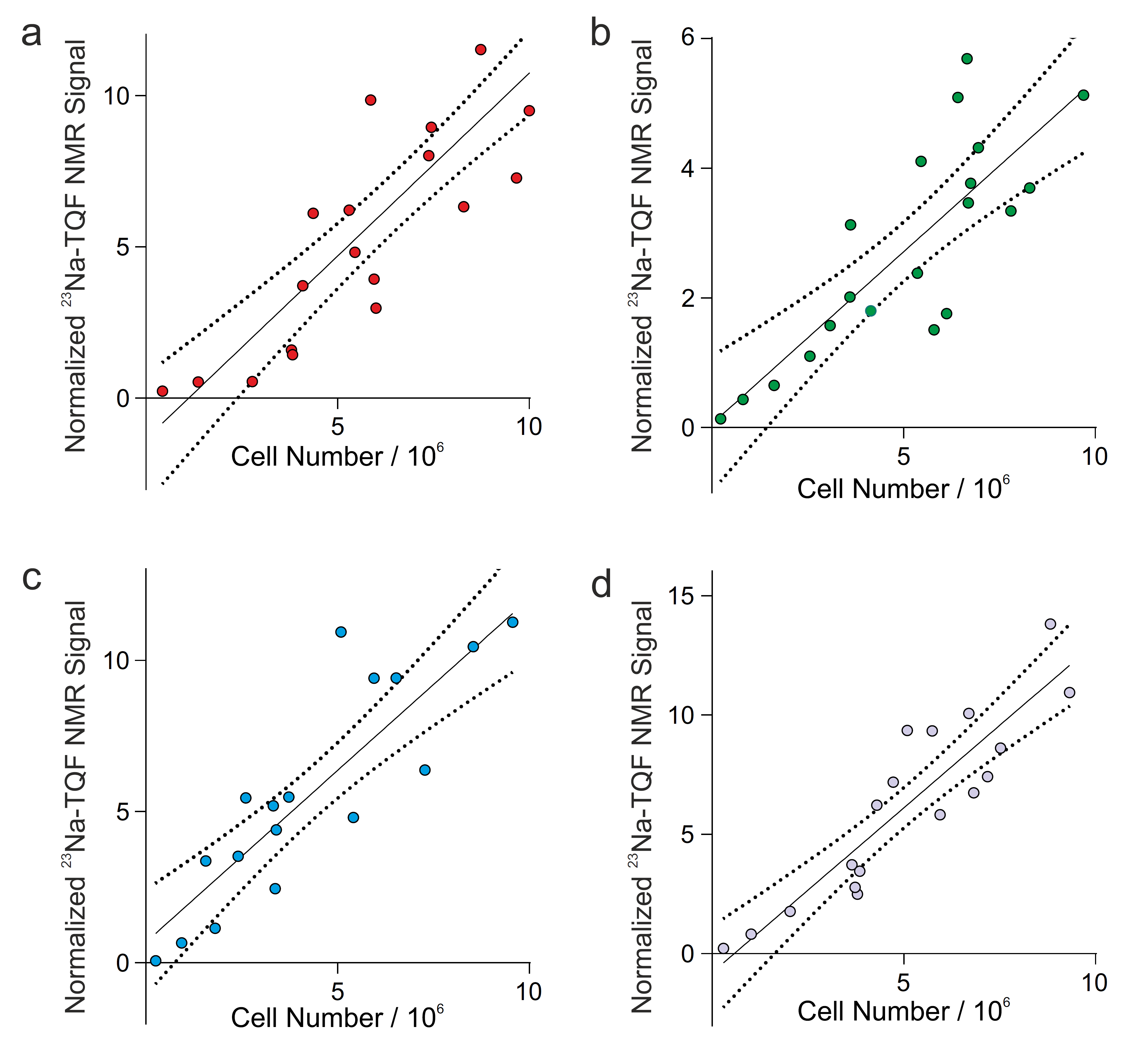


**Supplementary Figure S5. ^23^Na-TQF NMR signal positively correlates with cell number.** ^23^Na-TQF peak integrals normalized to the reference peak and plotted against cell number (× 10^6^). Linear regressions are plotted with 5% confidence intervals showing a statistically ‘good’ correlation: 4T1 (p < 0.0001, R squared = 0.804, n = 21), MDA-MB-231 (p < 0.0001, R squared = 0.670, n = 20), HCC1954 (p < 0.0001, R squared = 0.764, n = 18), MCF-7 (p < 0.0001, R squared = 0.815, n = 18).


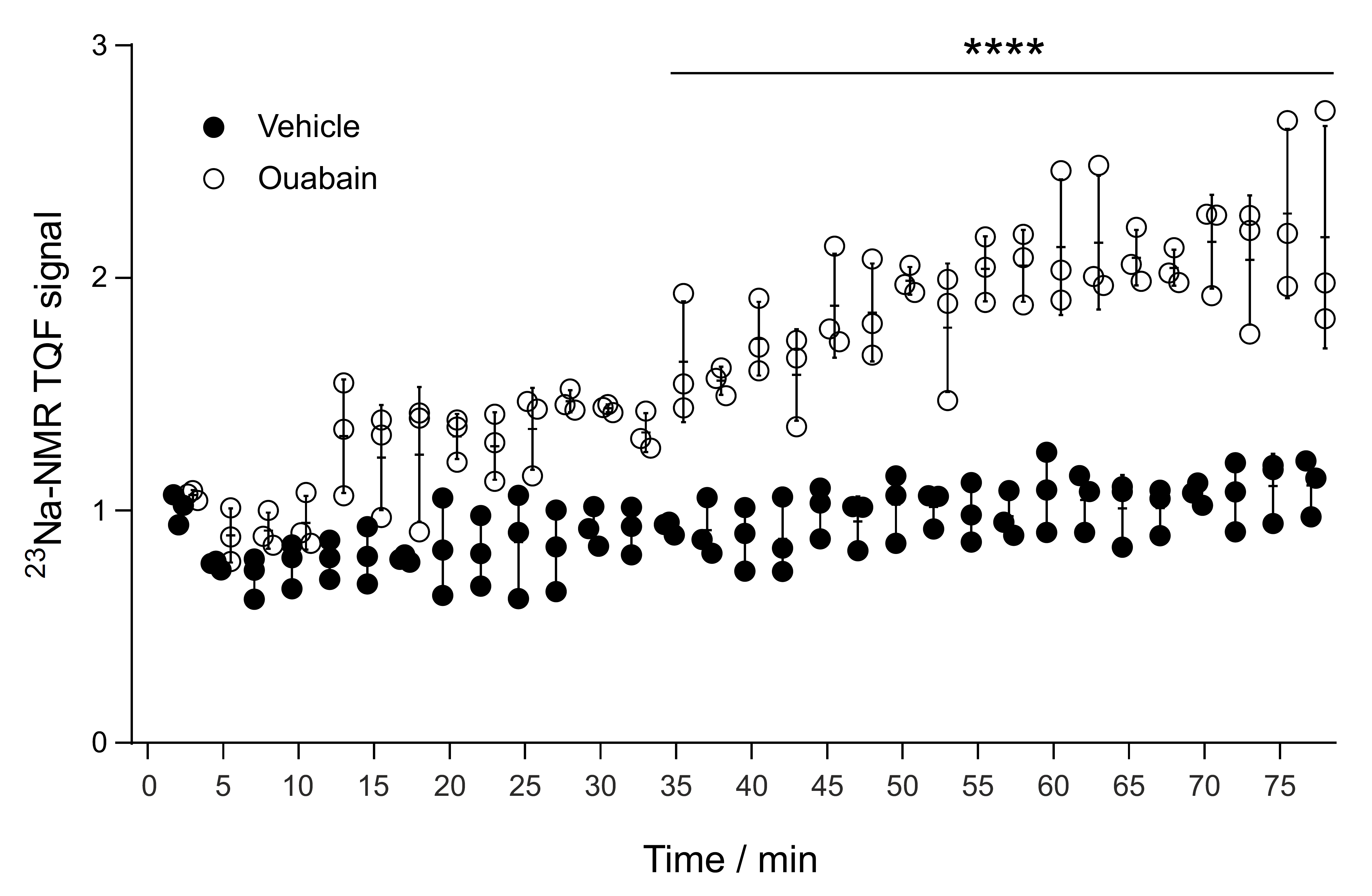


**Supplementary Figure S6. ^23^Na-TQF NMR signal increased in MDA-MB-231 cells following ouabain treatment.** Following resuspension in DMEM ± 1 µM ouabain, the intracellular sodium-ion signal was measured by ^23^Na-TQF NMR in MDA-MB-231 cells over the course of 1 h. Values were normalized to the ^23^Na-TQF signal before the addition of vehicle or ouabain (1.000). At the end of the 1 h investigation, the ^23^Na-TQF signal in vehicle treated cells was 1.11 ± 0.12 while ouabain treated cells increased to 2.17 ± 0.48 (n = 3). A two-way ANOVA with Sidak’s multiple comparisons was performed yielding p < 0.05; ****<0.0001.





**Supplementary Figure S7. Representative Seahorse XFe glycolytic stress test from 4T1 cells.** The first three time points correspond to baseline ECAR. Glycolytic rate was measured by subtracting the baseline from the maximum ECAR measured after addition of 10 mM glucose. Total glycolytic capacity was measured by subtracting the baseline from the maximum ECAR measured after addition of 1 μM oligomycin. Glycolytic reserve was calculated as the difference between the total glycolytic capacity and the glycolytic rate. The final addition of 100 mM 2-deoxy-D-glucose inhibits glycolysis so that ECAR returns to its baseline value.


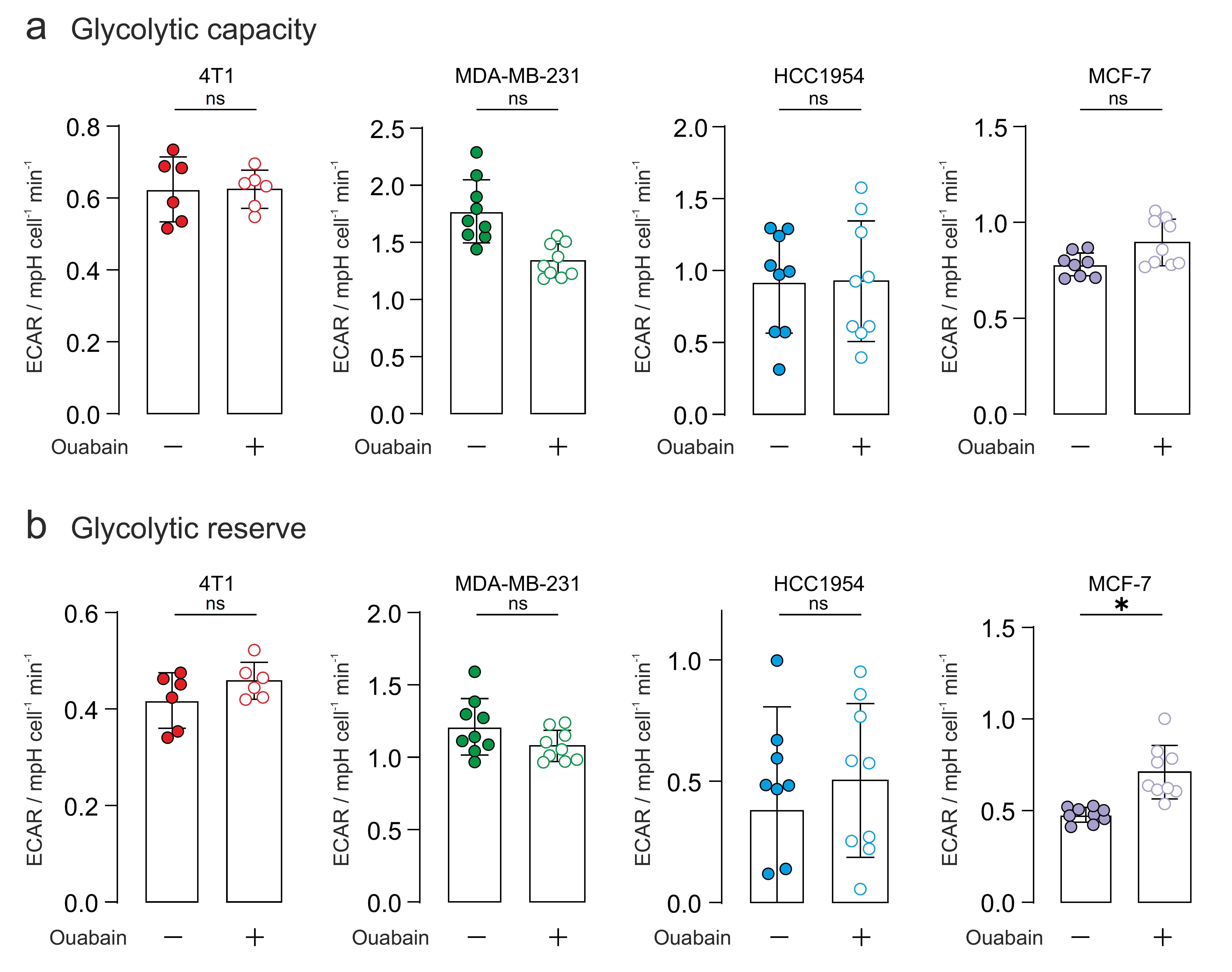


**Supplementary Figure S8. Quantification of the extracellular acidification rate.** (A) Glycolytic capacity and (B) glycolytic reserve measured from Seahorse XFe glycolytic stress test as defined in Supplementary Figure S6. (n = 3 biological repeats each with n = 3 technical repeats; significance was assessed using a nested unpaired t-test, ns p > 0.05, * p < 0.05).


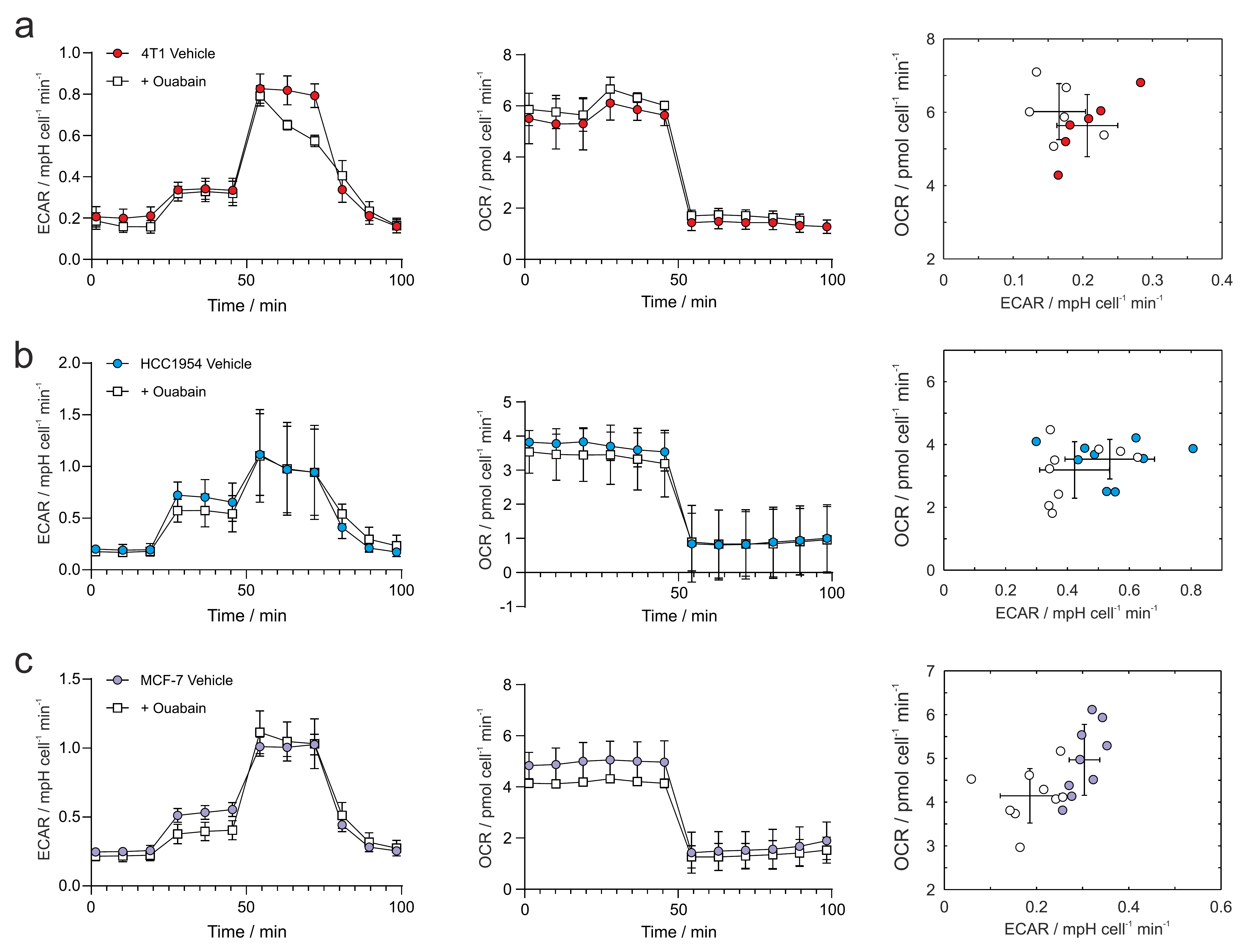


**Supplementary Figure S9. Extracellular acidification rate (ECAR) and oxygen consumption rates (OCR) measured during Seahorse XFe glycolytic stress tests.** Rows, (A) 4T1 cells, (B) HCC1954 cells, (C) MCF-7 cells. MDA-MB-231 time courses are given in Figure 2. ECAR data are shown in the left column which were measured simultaneously with the OCR data, middle column. Timings of the addition of 10 mM glucose, 1 μM oligomycin, and 100 mM 2-deoxy-D-glucose are shown in Supplemental Figure S6. Right column shows plots of the measured glycolytic rate vs OCR for the same samples plotted in Figure 2 (n = 3 biological repeats each with n = 3 technical repeats).


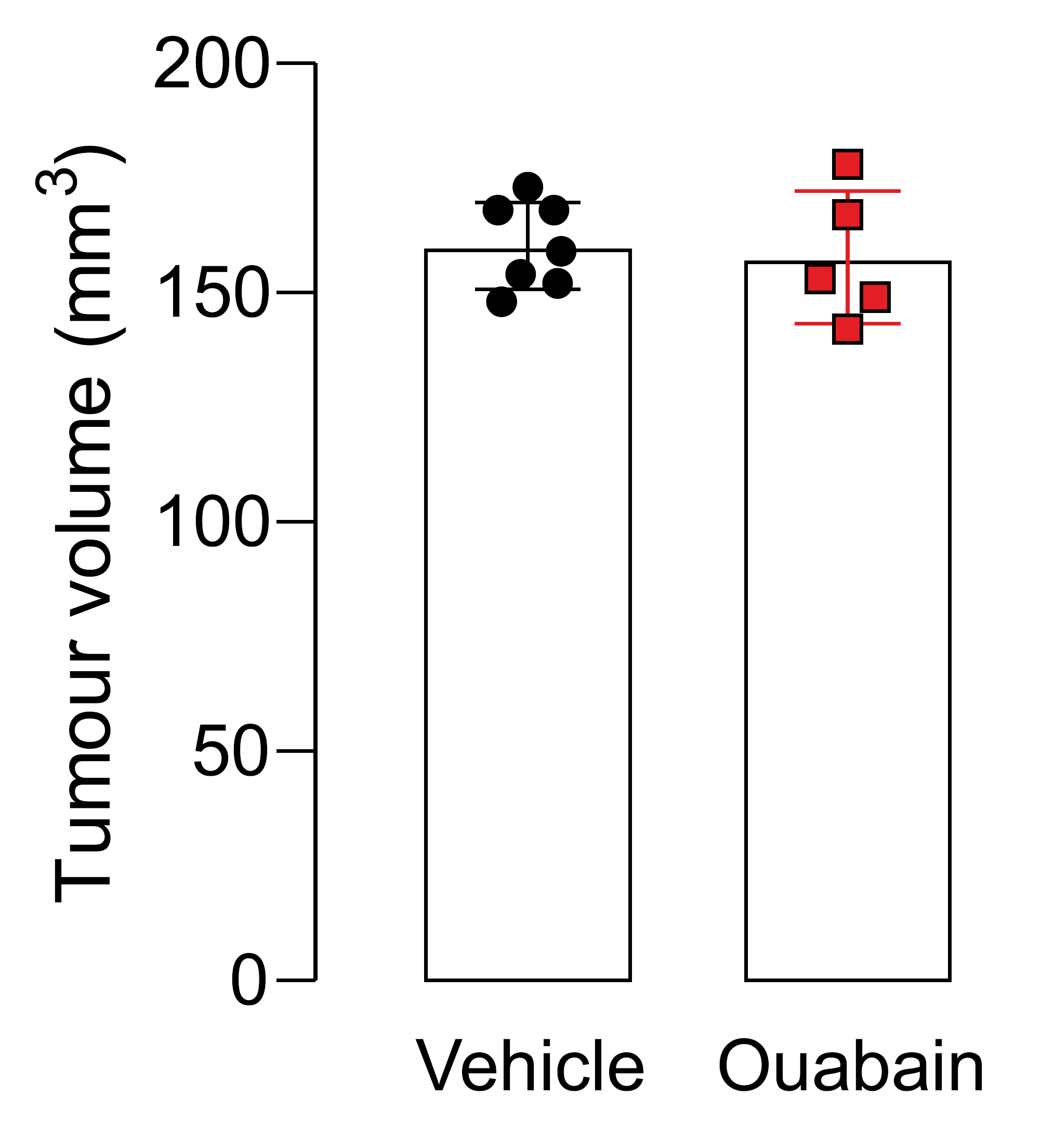


**Supplementary Figure S10. Tumour volumes at point of imaging.** Tumour growth was monitored daily by electronic calliper measurement with volume calculated using the following equation: volume (mm^3^) = ((π/6) × h × w × l), where h, w and l represent height, weight, and length, respectively. When tumours reached ~150 mm^3^ they were selected for imaging. The administration of vehicle ± ouabain was blinded until after PET reconstruction analysis. Tumour volumes for each group were comparable: vehicle, 160 ± 9.50 mm^3^ versus ouabain, 158 ± 14.5 mm^3^.


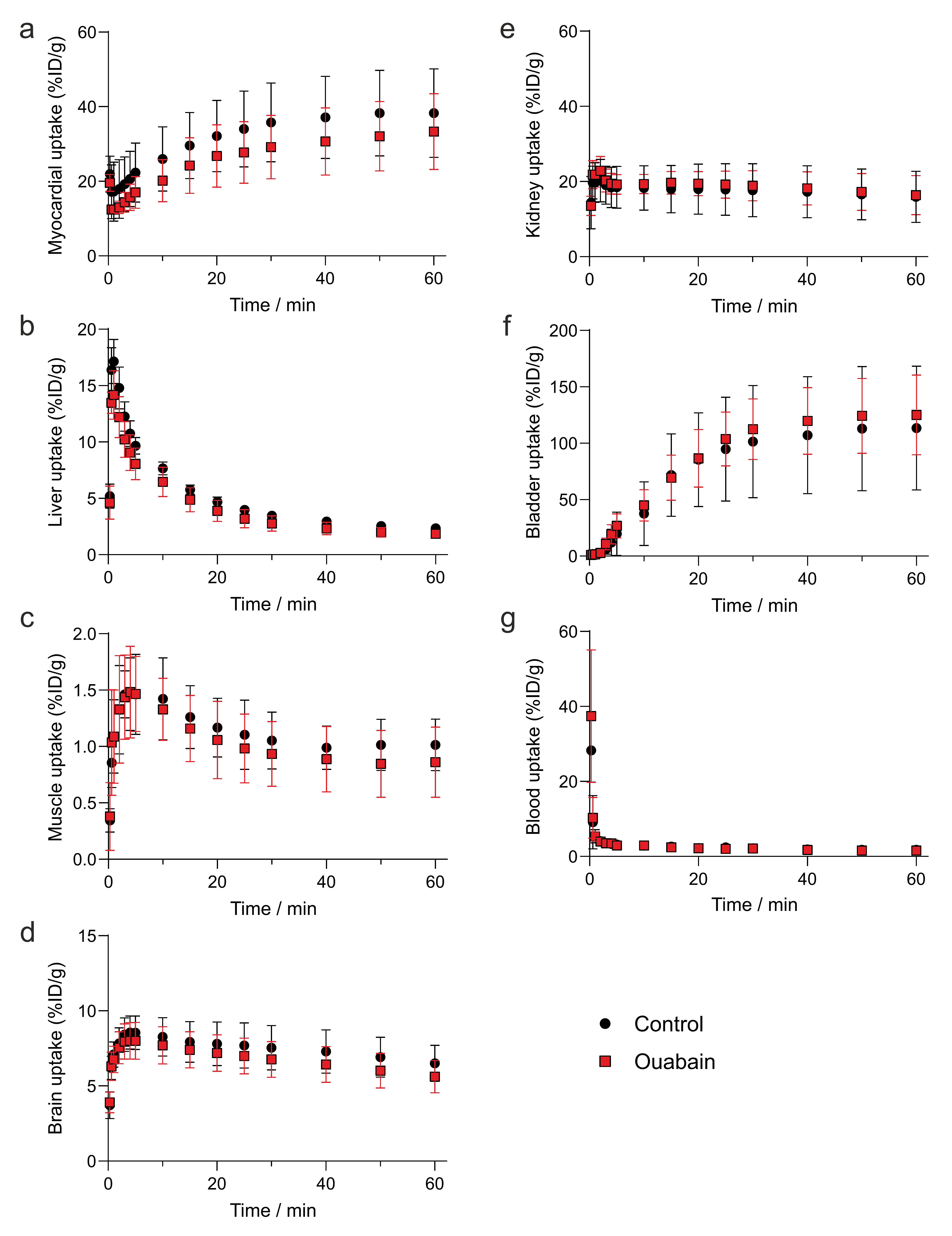


**Supplementary Figure S11. Time-activity curves of ^18^F-FDG in organs of interest.** Time activity curves reveal the whole-body biodistribution of ^18^F-FDG in vehicle (black, n = 7) *versus* ouabain treated groups (red, n = 5). The dynamic reconstruction method allowed for the tracing of ^18^F-FDG through the 1 h imaging protocol. Notable uptake is observed in the myocardium and bladder (via renal excretion). Rapid blood clearance and liver clearance is observed. There was no significant difference in ^18^F-FDG uptake observed in any of the tissues in the ouabain treated group *versus* the vehicle control.
